# Body composition is associated with postoperative complications in perihilar cholangiocarcinoma

**DOI:** 10.1002/cam4.6878

**Published:** 2024-01-01

**Authors:** Guanwu Wang, Anna Mantas, Lara R. Heij, Tarick M. Al‐Masri, Dong Liu, Daniel Heise, Sophia M. Schmitz, Steven W. M. Olde Damink, Tom Luedde, Sven A. Lang, Tom F. Ulmer, Ulf P. Neumann, Jan Bednarsch

**Affiliations:** ^1^ Department of Surgery and Transplantation University Hospital RWTH Aachen Aachen Germany; ^2^ University of Applied Science Aachen Aachen Germany; ^3^ Department of Surgery and Transplantation University Hospital Essen Essen Germany; ^4^ Department of Surgery Maastricht University Medical Centre (MUMC) Maastricht The Netherlands; ^5^ Department of Gastroenterology, Hepatology and Infectious Diseases Heinrich Heine University Duesseldorf Duesseldorf Germany

**Keywords:** body composition, cholangiocellular carcinoma, oncological outcome, perioperative complications

## Abstract

**Background:**

Perihilar cholangiocarcinoma (pCCA) is a malignant tumor of the hepatobiliary system which is still associated with a challenging prognosis. Postoperative complications play a crucial role in determining the overall prognosis of patients with pCCA. Changes in body composition (BC) have been shown to impact the prognosis of various types of tumors. Therefore, our study aimed to investigate the correlation between BC, postoperative complications and oncological outcome in patients with pCCA.

**Methods:**

All patients with pCCA who underwent curative‐intent surgery for pCCA between 2010 and 2022 were included in this analysis. BC was assessed using preoperative computed tomography and analyzed with the assistance of a 3D Slicer software. Univariate and multivariate binary logistic regression analyses were conducted to examine the relationship between BC and clinical characteristics including various measurements of postoperative complications and Cox regressions and Kaplan–Meier analysis to evaluate oncological risk factors in the study cohort.

**Results:**

BC was frequently altered in patients undergoing curative‐intent liver resection for pCCA (*n* = 204) with 52.5% of the patients showing obesity, 55.9% sarcopenia, 21.6% sarcopenic obesity, 48.5% myosteatosis, and 69.1% visceral obesity. In multivariate analysis, severe postoperative complications (Clavien‐Dindo ≥3b) were associated with body mass index (BMI) (Odds ratio (OR) = 2.001, *p* = 0.024), sarcopenia (OR = 2.145, *p* = 0.034), and myosteatosis (OR = 2.097, *p* = 0.017) as independent predictors. Furthermore, sarcopenia was associated with reduced overall survival (OS) in pCCA patients (sarcopenia vs. no‐sarcopenia, 21 months vs. 32 months, *p* = 0.048 log rank).

**Conclusions:**

BC is highly associated with severe postoperative complications in patients with pCCA and shows tendency to be associated impaired overall survival. Preoperative assessment of BC and interventions to improve BC might therefore be key to improve outcome in pCCA patients undergoing surgical therapy.

## 1 INTRODUCTION

Cholangiocarcinoma (CCA) is the second most common primary liver cancer after hepatocellular carcinoma (HCC), accounting for 3% of all gastrointestinal cancers.^1^ It is classified anatomically into intrahepatic (iCCA), perihilar (pCCA) or distal (dCCA) subtypes with pCCA being the most prevalent form of CCA.^1^ Surgery is considered as the primary treatment for pCCA.^2^ However, as major liver resection together with complex vascular and biliary reconstruction are commonly necessary in these patient, notable perioperative morbidity, and mortality rates up to 15% have been reported.^3^ A previously conducted meta‐analysis, of 4634 patients, revealed a 40% incidence of severe postoperative complications Clavien‐Dindo grade ≥3 after pCCA surgery and representative 90‐day mortality of 9%.^4^ Also, the cumulative postoperative complications following pCCA resection have a substantial impact on the long‐term survival of these patients.^5^

Cancer‐associated cachexia is a multifaceted condition characterized by changes in body composition (BC) including weight loss, skeletal muscle wasting (with or without adipose tissue depletion), and a gradual decline in physiological function.^6^ In patients with pCCA, preoperative skeletal muscle wasting and mass have been identified as prognostic factors for overall survival (OS).^7^, ^8^, ^9^ Postoperative complications have been identified as one of the most important elements affecting postoperative survival in pCCA patients.^5^ However, only one article had described an association between preoperative skeletal muscle mass exclusively and the incidence of infectious complications in patients with pCCA.^8^ Alterations in BC, including body mass index (BMI), sarcopenia, myosteatosis, visceral obesity, sarcopenic obesity, and visceral to subcutaneous adipose tissue ratio (VSR) serve as prognostic markers in a variety of clinical settings including the progression of oncological and liver disease.^10^ Alterations in BC have also been linked to unfavorable prognosis and reduced long‐term survival in various cancer types, including HCC, gastric cancer, and breast cancer.^11^, ^12^, ^13^

Nevertheless, the impact of fluctuations in BC on postoperative complications in patients diagnosed with pCCA remains poorly understood. Until now, only limited research has explored the potential significance of preoperative BC in predicting postoperative complications among patients with pCCA.^8^ In this study, we conducted a retrospective analysis in a large monocentric cohort to examine the correlation between preoperative BC characteristics and postoperative complications in individuals who underwent surgery for pCCA. We hypothesized that preoperative BC and the occurrence of postoperative complications and long‐term outcome are notably associated.

## 2 MATERIALS AND METHODS

### 2.1 Study population

We conducted a retrospective study of patients who underwent surgery for pCCA at the RWTH Aachen University Hospital between 2010 and 2022. Exclusion criteria were as follows: (1) patients did not have abdominal CT scans not older than 1 month prior resection, (2) individuals lacking images of the third lumbar vertebral level (L3), (3) patients who relied solely on alternative imaging modalities such as magnetic resonance imaging (MRI). As 10 patients were excluded due to the above‐mentioned criteria, the final cohort comprised a total of 204 patients with pCCA. Written informed consent was obtained from the study patients. This study adhered to the principles outlined in the Declaration of Helsinki and received approval from the Ethics Committee of RWTH Aachen University Hospital (EK 23‐269).

### 2.2 Evaluation of computed tomography imaging

A dual‐source CT scanner (Siemens Somatom Force, Siemens AG, Munich, Germany) was used for all CT scans. Technical parameters for CT imaging included a tube voltage of 120 kVp, a rotation speed of 0.5 s/rotation and a reconstruction thickness of 5 mm. All CT images underwent contrast enhancement with the images being acquired in the venous phase. Skeletal muscle and adipose tissue in single cross‐sectional CT images at the level of the third lumbar vertebra were semi‐automatically segmented using the 3D Slicer software platform version 4.1 and the BC module (https://www.slicer.org/) (L3). Attenuation values ranging from −29 to 150 Hounsfield Units (HU) were used to identify and quantify skeletal muscles. Muscle area at the L3 level included various muscles such as the psoas major, erector spinae, lumbar quadrate, transverse abdominis, external and internal obliques, and rectus abdominis. The skeletal muscle index (SMI), calculated by dividing skeletal muscle area (SMA) by the square of height (cm^2^/m^2^) was used to identify patients with sarcopenia. Visceral fat area (VFA) was assessed using attenuation values between −150 and −50 HU, while subcutaneous adipose tissue was identified using attenuation values between −190 and −30 HU. All measurements were performed by two blinded investigators (GW, JB). In that study, the intra‐rater and inter‐rater coefficient of variation for SMI was found to be 0.60% and 2.90%, 1.30% and 2.60% for VFA, and 1.20% and 2.40% for SM‐RA, respectively.

**FIGURE 1:**
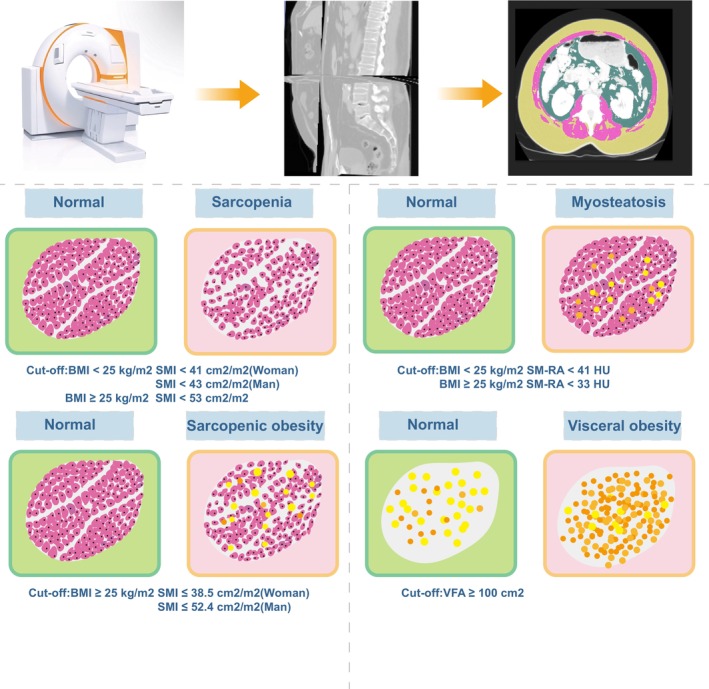
Post‐segmentation analysis of preoperative third lumbar spine CT in patients with perihilar cholangiocarcinoma for the identification of sarcopenia, myosteatosis, visceral obesity, and sarcopenic obesity. The corresponding regions were defined using the following attenuation values: Skeletal muscle region (purple), −29‐150 HU; subcutaneous fat region (yellow), −190 to −30 HU; visceral fat region (dark green), −150 to −50 HU. BMI, body mass index; VFA, visceral fat area.

Historically, a variety of cutoff values have been used for the assessment of BC.^14^ The definition of overweight or obesity was based on a BMI ≥25 kg/m^2^. Sarcopenia was diagnosed if the SMI was <41 cm^2^/m^2^ in women and < 43 cm^2^/m^2^ in men with BMI < 25 kg/m^2^, or if the SMI was <53 cm^2^/m^2^ with a BMI ≥25 kg/m^2^. Myosteatosis was diagnosed when SM‐RA was <41 HU for BMI < 25 kg/m2 or < 33 HU for BMI ≥25 kg/m^2^. VFA was considered high if ≥100 cm^2^. Sarcopenic obesity was diagnosed if SMI was ≤38.5 cm2/m2 for women and ≤ 52.4 cm^2^/m^2^ for men with BMI ≥25 kg/m^2^. VSR was divided into low VSR (<1.1) and high VSR (≥1.1) (Figure 1).

### 2.3 Statistical analysis

All statistical analyses were performed using SPSS software (IBM SPSS Statistics, version 26.0). Data are presented as numbers and percentages or median and interquartile ranges as data distribution might not be distributed normally in every subanalysis. Univariate and multivariate logistic regression analyses were performed to investigate the relationships between different clinical parameters in BC. Major complications were defined as any complications rated ≥3b according to the Clavien‐Dindo Scale. The Kaplan–Meier method was used to estimate overall survival (OS) and recurrence‐free survival (RFS). Cox regressions were performed to identify the prognostic factors associated with OS and RFS. Statistical significance was set at *p* < 0.05.

## 3 RESULTS

### 3.1 Patient characteristics

A total of 204 patients diagnosed with pCCA were enrolled in this study. The median age of the population was 68 years with most of the individuals being sassed as ASA III or higher (127/204, 62.3%). A variety of operative procedures with median operation time of 431 min was conducted. R0 resection was achieved in 164 (80.4%) of the patients. In terms of postoperative complications, measures for overall complications according to the Clavien‐Dindo‐Scale, infectious complications according to the Clavien‐Dindo‐Scale as well as liver failure, postoperative hemorrhage, and bile leakage according to the International Study Group of Liver Surgery (ISGLS) definitions were assessed.^15^, ^16^, ^17^, ^18^ Regarding, overall complications, 120 (58.8%) had a displayed complications Clavien‐Dindo grade ≤ 3A and 84 (41.2%) grade ≥ 3B. The perioperative mortality rate was 15.7% (32/206). More details regarding the study cohort are displayed Table 1.

**TABLE 1:** Patients characteristics.

Variables	*n* = 204
**Demographics**	
Gender, M/F (%)	139(66.5)/70(33.5)
Age (years)	68(58–74)
ASA, *n* (%)	
I	8(3.9)
II	69(33.8)
III	114(55.9)
IV	13(6.4)
V	0(0)
Bismuth type, *n* (%)	
I	11(5.4)
II	27(13.2)
IIIa	59(28.9)
IIIb	50(24.5)
IV	57(27.9)
Cholangitis, *n* (%)	65(31.9)
Portal vein embolization, *n* (%)	71(34.8)
Preoperative Chemotherapy, *n* (%)	10(4.9)
**Clinical chemistry**	
AST (U/l)	45 (34–86)
ALT (U/l)	58 (36–113)
Albumin (g/L)	3.8 (3.4–4.0)
AP, U/L	267 (159–424)
CA19‐9 (U/ml)	99 (35–329)
CRP (mg/l)	12.0 (6.0–35.7)
GGT (U/l)	424 (188–761)
Hemoglobin (g/dl)	12.1 (11.0–13.3)
INR	1.03 (0.97–1.11)
Platelet count (/nl)	294 (228–388)
Prothrombin time (%)	96 (85–105)
Total bilirubin (mg/dl)	1.1 (0.6–2.8)
**Operative Data**	
Intraoperative PRBC, *n* (%)	101 (49.5)
Intraoperative FFP, *n* (%)	109 (53.4)
Operative time (min)	431 (374–500)
Operative procedure, *n* (%)	
Hemihepatectomy	54 (26.4)
Extended hemihepatectomy	91 (44.6)
Trisectionectomy	34 (16.7)
Hepatoduodenoectomy	13 (6.4)
ALPPS	2 (1.0)
Others	10 (4.9)
**Pathological examination**	
LVI, *n* (%)	45 (22.1)
MVI, *n* (%)	62 (30.4)
R1 resection, *n* (%)	40 (19.6)
pT category *n* (%)	
1	17 (8.4)
2	116 (56.9)
3	50 (24.5)
4	19 (9.3)
pN category, *n* (%)	
N0	115 (56.4)
N1	88 (43.1)
Tumor grading, *n* (%)	
G1	8 (3.9)
G2	135 (66.2)
G3	52 (25.5)
G4	1 (0.5)
**Postoperative Data**	
Intensive care, days	1 (1–3)
Hospitalization, days	19 (12–35)
Postoperative complications, *n* (%)	
No complications	33 (16.2)
Clavien‐Dindo I	12 (5.9)
Clavien‐Dindo II	39 (19.1)
Clavien‐Dindo IIIa	36 (17.6)
Clavien‐Dindo IIIb	34 (16.7)
Clavien‐Dindo IVa	11 (5.4)
Clavien‐Dindo IVb	7 (3.4)
Clavien‐Dindo V	32 (15.7)
Liver failure, *n* (%)	
None	167 (81.9)
Grade A	18 (8.8)
Grade B	11 (5.4)
Grade C	8 (3.9)
Infection Clavien‐Dindo	
No complications	69 (33.8)
Clavien‐Dindo I	2 (1.0)
Clavien‐Dindo II	44 (21.6)
Clavien‐Dindo IIIa	41 (20.1)
Clavien‐Dindo IIIb	13 (6.4)
Clavien‐Dindo IVa	22 (10.8)
Clavien‐Dindo IVb	22 (10.8)
Clavien‐Dindo V	7 (3.4)
Bile leakage, *n* (%)	
None	155 (76.0)
Grade A	14 (6.9)
Grade B	19 (9.3)
Grade C	16 (7.8)
Hemorrhage, *n* (%)	
None	165 (80.9)
Grade A	9 (4.4)
Grade B	6 (2.9)
Grade C	24 (11.8)
**Oncologic Data**	
Adjuvant chemotherapy, *n* (%)	54 (26.5)
Recurrence, *n* (%)	78 (38.2)
Median RFS, months (95% CI)	35 (22–48)
Median OS, months (95% CI)	27 (19–35)
**Body composition**	
BMI (kg/m2)	25.28 (22.87–28.36)
Visceral_fat area (cm^2^)	142.37 (84.26–225.50)
SMI (cm^2^/m^2^)	46.06 (39.76–53.47)
SM‐RA (HU)	37.00(30.23–43.46)
Obesity, *n* (%)	107 (52.5)
Sarcopenia, *n* (%)	114 (55.9)
Myosteatosis, *n* (%)	99 (48.5)
Visceral obesity, *n* (%)	107 (52.5)
Sarcopenic obesity, *n* (%)	44 (21.6)
High VSR, *n* (%)	66 (32.4)

*Note*: Data presented as median and interquartile range if not noted otherwise.

Abbreviations: ALPPS, associating liver partition with portal vein ligation for staged hepatectomy; ALT, alanine aminotransferase; ASA, American Society of Anesthesiology; AST, aspartate aminotransferase; AP, alkaline phosphatase; BMI, body mass index; CRP, C‐reactive protein; F, female; FFP, fresh frozen plasma; GGT, gamma‐glutamyl transferase; INR, international normalized ratio; LVI, lymph vascular invasion; M, male; MVI, microvascular invasion; OS, overall survival; PRBC, packed red blood cells; RFS, recurrence‐free survival; SMI, skeletal muscle index; SM‐RA, skeletal muscle radiation attenuation; VSR, visceral to subcutaneous adipose tissue ratio.

### 3.2 Associations of body composition to clinical characteristics and complications

In the study cohort, 107 patients (52.5%) presented with obesity, 114 patients (55.9%) were classified as sarcopenic, 99 patients (48.5%) exhibited myosteatosis, 141 patients (69.1%) displayed visceral obesity, 44 patients (21.6%) demonstrated sarcopenic obesity and 66 patients (32.4%) presented with a high VSR (Table 1). To assess the relationship between these BC characteristics and clinical features including postoperative complications, univariate and multivariate logistic regression analyses were conducted. To ensure the validity of results all above mentioned measures for complications were included in the analysis among other known clinical variables.

The detailed results of the analysis are displayed in Table 2 and Table S1. The brief results of the multivariate analysis were as followed: Individuals with obesity showed low levels of alkaline phosphatase (odds ratio, (OR) = 0.081, *p* = 0.019), reduced lymph node involvement (OR = 0.507, *p* = 0.025) and an increased likelihood of experiencing severe postoperative complications (OR = 2.001, *p* = 0.024). Patients with sarcopenia were more likely to be female (OR = 8.102, *p*<0.001) and did also experience more serious post‐operative complications (OR = 2.145, *p* = 0.034).

**TABLE 2:** Synopsis of the univariate analysis and multivariate analysis of clinical and pathological data associated with body composition in perihilar cholangiocarcinoma.

Outcome	Descriptives		Univariate analysis		Multivariate analysis	
BMI (kg/m^2^)	<25 (*n* = 97)	≥25 (*n* = 107)	OR (95% CI)	*p* value	OR (95% CI)	*p* value
AP, U/L (≤100/ >100(%); ref = ≤100)	2(2.1)/91(93.8)	10(9.3)/94(87.9)	0.207(0.044–0.969)	0.045	**0.081(0.010–0.660)**	**0.019**
pN category (N0/N1(%); ref = N0)	47(48.5)/49(50.5)	68(63.6)/39(36.4)	0.550(0.314–0.964)	0.037	**0.507(0.279–0.920)**	**0.025**
Postoperative complications, Clavien‐Dindo ((0/I/II/IIIa)/(IIIa/IV/V) (%); ref = 0/I/II/IIIa)	64(66.0)/33(34.0)	56(52.3)/51(47.7)	1.766(1.003–3.111)	0.049	**2.001(1.097–3.653)**	**0.024**
Liver failure (No/Yes(%);ref = No)	75(77.3)/22(22.7)	92(86.0)/15(14.0)	0.556(0.270–1.146)	0.112		
Bile leak (No/Yes(%);ref = No)	76(78.4)/21(21.6)	79(73.8)/28(26.2)	1.283(0.671–2.451)	0.451		
Hemorrhage (No/Yes(%);ref = No)	79(81.4)/18(18.6)	86(80.4)/21(19.6)	1.072(0.532–2.158)	0.846		
Infection Clavien‐Dindo ((0/I/II/IIIa)/(IIIa/IV/V) (%);ref = 0/I/II/IIIa)	77(79.4)/19(19.6)	79(73.8)/28(26.2)	1.436(0.741–2.784)	0.283		
Sarcopenia	No (*n* = 90)	Yes (*n* = 114)	OR (95% CI)	*p* value	OR (95% CI)	*p* value
Sex (male/female(%); ref = male)	78(86.7)/12(13.3)	58(50.9)/56(49.1)	6.276(3.085–12.766)	<0.001	**8.102(3.511–18.695)**	**<0.001**
ALT, U/L (≤40/ >40(%); ref = ≤40)	16(17.8)/58(64.4)	36(31.6)/59(51.8)	0.452(0.226–0.903)	0.024	0.530(0.248–1.135)	0.102
Postoperative complications Clavien‐Dindo ((0/I/II/IIIa)/(IIIa/IV/V) (%); ref = 0/I/II/IIIa)	37(41.1)/29(32.2)	37(32.5)/44(38.6)	1.800(1.016–3.189)	0.044	**2.145(1.057–4.350)**	**0.034**
Liver failure (No/Yes(%);ref = No)	75(83.3)/15(16.7)	92(80.7)/22(19.3)	1.196(0.580–2.465)	0.628		
Bile leak (No/Yes(%);ref = No)	71(78.9)/19(21.1)	84(73.7)/30(26.3)	1.355(0.693–2.571)	0.388		
Hemorrhage (No/Yes(%);ref = No)	73(81.1)/17(18.9)	92(80.7)/22(19.3)	1.027(0.508–2.075)	0.941		
Infection Clavien‐Dindo ((0/I/II/IIIa)/(IIIb/IV/V) (%);ref = 0/I/II/IIIa)	74(82.2)/15(16.7)	82(71.9)/32(28.1)	1.925(0.966–3.835)	0.062		
Myosteatosis	No (*n* = 105)	Yes (*n* = 99)	OR (95% CI)	*p* value	OR (95% CI)	*p* value
Sex (male/female(%); ref = male)	78(76.3)/27(25.7)	58(58.6)/41(41.4)	2.042(1.129–3.694)	0.018	**2.254(1.197–4.242)**	**0.012**
Age (≤65/ >65 years(%); ref = ≤65)	56(53.3)/49(46.7)	31(31.3)/68(68.7)	2.507(1.415–4.443)	0.002	**2.691(1.473–4.916)**	**0.001**
Cholangitis (No/Yes (%); ref = No)	79(75.2)/26(24.8)	60(60.6)/39(39.4)	1.975(1.085–3.595)	0.026	**1.965(1.039–3.719)**	**0.038**
Postoperative complications Clavien‐Dindo ((0/I/II/IIIa)/(IIIa/IV/V) (%); ref = 0/I/II/IIIa)	70(66.7)/35(33.3)	50(50.5)/49(49.5)	1.960(1.113–3.450)	0.020	**2.097(1.143–3.847)**	**0.017**
Liver failure (No/Yes(%);ref = No)	87(82.9)/18(17.1)	80(80.8)/19(19.2)	1.148(0.563–2.341)	0.704		
Bile leak (No/Yes(%);ref = No)	80(76.2)/25(23.8)	75(75.8)/24(24.2)	1.024(0.539–1.947)	0.942		
Hemorrhage (No/Yes(%);ref = No)	89(84.8)/16(15.2)	76(76.8)/23(23.2)	1.683(0.830–3.416)	0.149		
Infection Clavien‐Dindo ((0/I/II/IIIa)/(IIIa/IV/V) (%);ref = 0/I/II/IIIa)	83(79.0)/21(20.0)	73(73.7)/26(26.3)	1.408(0.731–2.711)	0.307		
Visceral obesity (VFA, cm^2^)	<100 (*n* = 63)	≥100 (*n* = 141)	OR (95% CI)	*p* value	OR (95% CI)	*p* value
Postoperative complications Clavien‐Dindo ((0/I/II/IIIa)/(IIIa/IV/V) (%);ref = 0/I/II/IIIa)	40(63.5)/23(36.5)	80(56.7)/61(43.3)	1.326(0.719–2.444)	0.366		
Liver failure (No/Yes(%);ref = No)	47(74.6)/16(25.4)	120(85.1)/21(14.9)	0.514(0.247–1.070)	0.075		
Bile leak (No/Yes(%);ref = No)	52(82.5)/11(17.5)	103(73.0)/38(27.0)	1.744(0.824–3.690)	0.146		
Hemorrhage (No/Yes(%);ref = N o)	50(79.4)/13(20.6)	115(81.6)/26(18.4)	0.870(0.413–1.830)	0.713		
Infection Clavien‐Dindo ((0/I/II/IIIa)/(IIIa/IV/V) (%);ref = 0/I/II/IIIa)	48(76.2)/14(22.2)	108(76.6)/33(23.4)	1.048(0.514–2.134)	0.898		
Sarcopenic obesity	No (*n* = 160)	Yes(*n* = 44)	OR (95% CI)	*p* value	OR (95% CI)	*p* value
Sex (male/female(%); ref = male)	100(62.5)/60(37.5)	36(81.8)/8(18.2)	0.370(0.161–0.850)	0.019	**0.170(0.056–0.519)**	**0.002**
AST, U/L (≤40/ >40(%); ref = ≤40)	61(38.1)/95(59.4)	25(56.8)/19(43.2)	0.488(0.248–0.961)	0.038	0.550(0.192–1.576)	0.266
ALT, U/L (≤40/ >40(%); ref = ≤40)	34(21.3)/100(62.5)	18(40.9)/17(38.6)	0.321(0.149–0.693)	0.004	**0.248(0.102–0.604)**	**0.002**
pN category (N0/N1(%);ref = N0)	81(50.6)/78(48.8)	34(77.3)/10(22.7)	0.305(0.141–0.660)	0.003	**0.307(0.123–0.767)**	**0.012**
Tumor grading ((G1/G2)/(G3/G4) (%);ref = G1/G2)	104(65.0)/48(30.0)	39(88.6)/5(11.4)	0.278(0.103–0.749)	0.011	**0.248(0.076–0.808)**	**0.021**
Postoperative complications Clavien‐Dindo ((0/I/II/IIIa)/(IIIa/IV/V) (%);ref = 0/I/II/IIIa)	96(60.0)/64(40.0)	24(54.5)/20(45.5)	1.250(0.638–2.449)	0.515		
Liver failure (No/Yes(%);ref = No)	131(81.9)/29(18.1)	36(81.8)/8(18.2)	1.004(0.423–2.385)	0.993		
Bile leak (No/Yes(%);ref = No)	122(76.3)/38(23.8)	33(75.0)/11(25.0)	1.070(0.494–2.319)	0.864		
Hemorrhage (No/Yes(%);ref = No)	130(81.3)/30(18.8)	35(79.5)/9(20.5)	1.114(0.484–2.564)	0.799		
Infection Clavien‐Dindo ((0/I/II/IIIa)/(IIIa/IV/V) (%);ref = 0/I/II/IIIa)	123(76.9)/36(22.5)	33(75.0)/11(25.0)	1.139(0.524–2.477)	0.743		
VSR	Low VSR(*n* = 138)	High VSR(*n* = 66)	OR (95% CI)	*p*‐value	OR (95% CI)	*p*‐value
Sex (male/female(%); ref = male)	75(54.3)/63(45.7)	61(92.4)/5(7.6)	0.098(0.037–0.258)	**<0.001**	**0.075(0.027–0.206)**	**<0.001**
Age (≤65/ >65 years(%); ref = ≤65)	66(47.8)/72(52.2)	21(31.8)/45(68.2)	1.964(1.061–3.638)	**0.032**	**2.439(1.213–4.904)**	**0.012**
ASA ((I/II)/(III/IV) (%);ref = I/II)	59(42.8)/79(57.2)	18(27.3)/48(72.7)	1.992(1.052–3.770)	**0.034**	**2.225(1.089–4.549)**	**0.028**
Postoperative complications Clavien‐Dindo ((0/I/II/IIIa)/(IIIa/IV/V) (%);ref = 0/I/II/IIIa)	79(57.2)/59(42.8)	41(62.1)/25(37.9)	0.816(0.448–1.489)	0.508		
Liver failure (No/Yes(%);ref = No)	112(81.2)/26(18.8)	55(83.3)/11(16.7)	0.862(0.397–1.871)	0.706		
Bile leak (No/Yes(%);ref = No)	107(77.5)/31(22.5)	48(72.7)/18(27.3)	1.294(0.660–2.538)	0.453		
Hemorrhage (No/Yes(%);ref = No)	105(76.1)/33(23.9)	60(90.9)/6(9.1)	0.318(0.126–0.803)	**0.015**	**0.274(0.101–0.742)**	**0.011**
Infection Clavien‐Dindo ((0/I/II/IIIa)/(IIIa/IV/V) (%);ref = 0/I/II/IIIa)	101(73.2)/37(26.8)	55(83.3)/10(15.2)	0.496(0.229–1.074)	0.075		

*Note*: The table only shows variables with *p* value <0.05 in the univariate analysis and all measures of postoperative complications as this was one of the key research questions. The full results of each univariate analysis are displayed in Table S1. Variables displaying a *p* value <0.05 in the univariate analysis were transferred into a multivariable Logistic regression model. Bold values display significance (*p* < 0.05).

Abbreviations: ALT, alanine aminotransferase; AP, Alkaline phosphatase; AST, aspartate aminotransferase; BMI, body mass index; OR, odds ratio; VSR, visceral to subcutaneous adipose tissue ratio.

In the context of myosteatosis, multifactorial analysis revealed that patients were more likely to be female (OR = 2.254, *p* = 0.012), elderly (OR = 2.691, *p* = 0.001), showed preoperative cholangitis (OR = 1.965, *p* = 0.038) and did also experience more serious postoperative complications (OR = 2.145, *p* = 0.034). Individuals with sarcopenic obesity were more likely to be male (OR = 0.170, *p* = 0.002), show low expression of ALT (OR = 0.248, *p* = 0.002), were less likely to have lymph node metastases (OR = 0.307, *p* = 0.012) and showed more differentiated tumor grades (OR = 0.248, *p* = 0.021). A higher VSR was significantly associated with male patients (OR = 0.075, *p* < 0.001), elderly patients (OR = 2.439, *p* = 0.012), higher ASA grades (OR = 2.225, *p* = 0.028), and a decreased probability of postoperative hemorrhage (OR = 0.274, *p* = 0.011). For visceral obesity, no association between clinical and laboratory features were assessed.

### 3.3 Kaplan–Meier analysis

**FIGURE 2:**
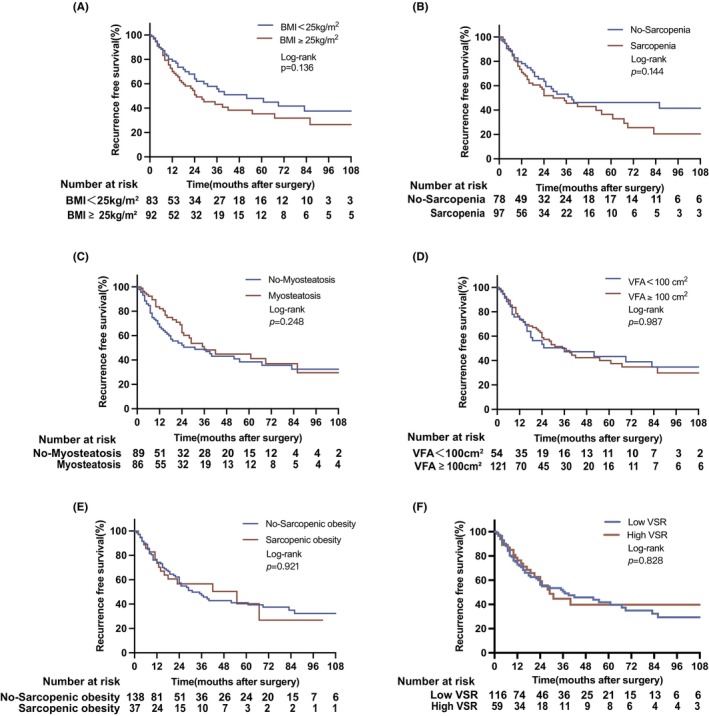
Recurrence‐free in relation to body composition characteristics in patients with perihilar cholangiocarcinoma. RFS for (A) BMI (BMI<25 kg/m^2^ vs. BMI≥25 kg/m^2^: 52 vs. 25 months), (B) sarcopenia (no‐sarcopenia vs sarcopenia: 29 vs. 39 months), (C) myosteatosis (no‐myosteatosis vs. myosteatosis: 31 vs. 36 months), (D) visceral obesity (no‐visceral obesity vs. visceral obesity: 36 vs. 35 months) and (E) sarcopenic obesity (no‐sarcopenic obesity vs. sarcopenic obesity: 31 vs. 42 months), (F) VSR (low VSR vs. high VSR: 37 vs. 29 months). BMI, body mass index; OS, over survival; RFS, recurrence free survival; VSR, visceral to subcutaneous adipose tissue ratio.

**FIGURE 3:**
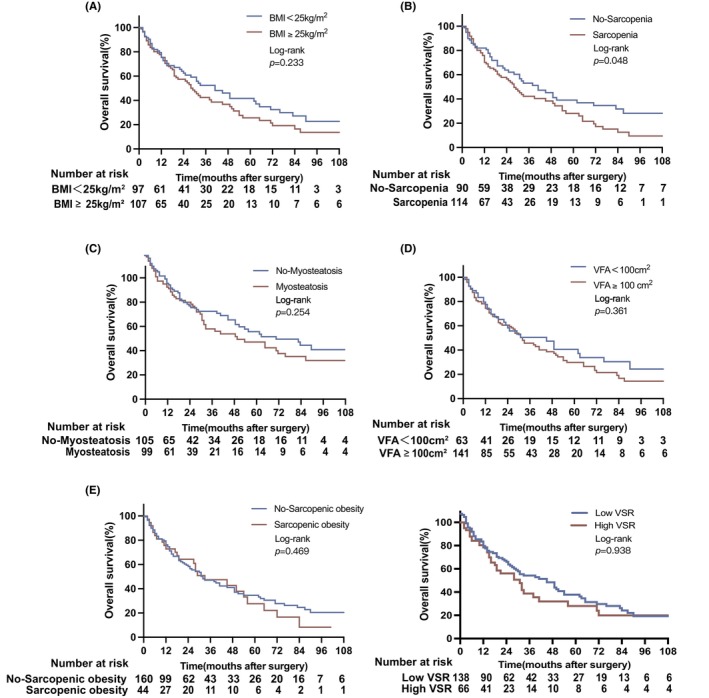
Overall survival in relation to body composition characteristics in patients with perihilar cholangiocarcinoma. OS for (A) BMI (BMI<25 kg/m^2^ vs. BMI≥25 kg/m^2^: 31 vs. 25 months), (B) sarcopenia (no‐sarcopenia vs sarcopenia: 31 vs. 21 months), (C) myosteatosis (no‐myosteatosis vs. myosteatosis: 25 vs. 27 months), (D) visceral obesity (no‐visceral obesity vs. visceral obesity: 25 vs. 27 months) and (E) sarcopenic obesity (no‐sarcopenic obesity vs. sarcopenic obesity: 25 vs. 28 months), (F) VSR (low VSR vs. high VSR:39 vs. 31 months). BMI, body mass index; OS, over survival; RFS, recurrence free survival; VSR, visceral to subcutaneous adipose tissue ratio.

After median follow‐up of 75 months, the study cohort showed a median RFS and OS of was 35 months and 27 months, respectively. For RFS, no significant differences in the study cohort were detected regarding BC (obese vs. nonobese: 25 vs. 52 months, *p* = 0.136; sarcopenia vs. no sarcopenia: 29 vs. 39 months, *p* = 0.144; myosteatosis vs. no myosteatosis: 36 vs. 31 months, *p* = 0.248; visceral obesity vs. no visceral obesity: 35 vs. 36 months, *p* = 0.987; sarcopenic obesity vs. no sarcopenic obesity: 42 vs. 31 months, *p* = 0.921; low VSR vs. high VSR: 37 vs. 29 months, *p* = 0.828) (Figure 2). It should be noted that longer RFS than OS is due to the notable number of perioperative deaths (15.7% [32/206]) which were part of the OS but not RFS analysis. For OS, patients with sarcopenia exhibited significantly lower OS (21 months), in comparison to non‐sarcopenic patients (32 months, *p* = 0.048, log‐rank test), while no other differences were observed for BC (obese vs. nonobese: 25 vs. 31 months, *p* = 0.233; myosteatosis vs. no myosteatosis: 27 vs. 25 months, *p* = 0.254; visceral obesity vs. no visceral obesity: 27 vs. 25 months, *p* = 0.361; sarcopenic obesity vs. no sarcopenic obesity: 28 vs. 25 months, *p* = 0.469; low VSR vs. high VSR: 39 vs. 31 months, *p* = 0.938) (Figure 3).

**FIGURE 4:**
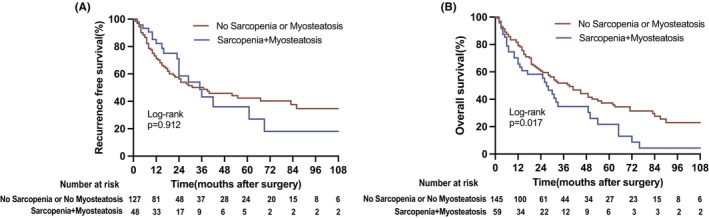
Overall and recurrence‐free survival with respect to the combination of sarcopenia and myosteatosis. (A) Recurrence‐free survival in patients with sarcopenia and myosteatosis (median RFS, no sarcopenia or no myosteatosis vs. sarcopenia and myosteatosis: 37 vs. 35 months). (B) Overall survival in patients sarcopenia and myosteatosis (median OS, no sarcopenia or no myosteatosis vs. sarcopenia and myosteatosis: 39 vs. 27 months). OS, overall survival. RFS, recurrence‐free survival.

Patients were further stratified for survival analysis based on the concurrent presence or absence of sarcopenia and myosteatosis. Compared to other patients, those with both conditions had a significantly shorter OS (27 months vs. 39 months, *p* = 0.017, log‐rank test). However, there was no statistically significant difference in RFS between the rest of the cohort and patients with both conditions (37 months vs. 35 months, *p* = 0.916, log‐rank test) (Figure 4).

### 3.4 Survival analysis by Cox regressions

To investigate the relationship between BC and clinical parameters with oncological outcomes, univariate and multivariate Cox regression analyses were conducted.

In the univariate analysis of our study, several variables were found to be significantly correlated with RFS. These variables included hemoglobin (*p* = 0.036), intraoperative PRBC transfusion (*p* < 0.001), intraoperative FFP transfusion (*p* = 0.001), operative time (*p* = 0.042), lymphovascular invasion (LVI, *p* < 0.001), R1 resection status (*p* < 0.001), T category (*p* < 0.001), N category (*p* < 0.001), tumor grade (*p* < 0.001), duration of hospitalization (*p* = 0.030), bile leak (*p* < 0.001), and infectious complications (*p* = 0.017). For the multivariate Cox regression model, we included all variables that showed *p* < 0.05 in the univariate analysis. After adjusting for other factors, the independent predictors of RFS were identified as intraoperative FFP transfusion (HR = 2.759, *p* < 0.001), LVI (HR = 2.193, *p* = 0.006), T category (HR = 1.864, *p* = 0.010), N category (HR = 1.831, *p* = 0.023), tumor grade (HR = 1.911, *p* = 0.014) and bile leak (HR = 2.538, *p* < 0.001) (Table 3).

**TABLE 3:** Analysis of recurrence‐free survival and overall survival in perihilar cholangiocarcinoma.

	Recurrence free survival				Overall survival			
	Univariable analysis		Multivariable analysis		Univariable analysis		Multivariable analysis	
Variables	HR(95%Cl)	*p* value	HR(95%Cl)	*p* value	HR(95%Cl)	*p* value	HR(95% Cl)	*p* value
Sex (Male = 1)	1.039(0.653–1.655)	0.871			1.046(0.730–1.497)	0.808		
Age, years (≤ 65 = 1)	0.831(0.535–1.293)	0.412			1.289(0.910–1.825)	0.153		
ASA (I/II =1)	1.177(0.747–1.854)	0.483			1.479(1.031–2.121)	0.033	**2.033(1.264–3.272)**	**0.003**
Cholangitis (No = 1)	1.468(0.914–2.357)	0.112			1.542(1.079–2.202)	0.017	1.164(1.021–2.610)	0.527
PVE (No =1)	0.959(0.601–1.532)	0.862			1.083(0.761–1.539)	0.658		
Neoadjuvant therapy (No =1)	0.484(0.119–1.970)	0.311			0.790(0.323–1.932)	0.605		
AST, U/l (≤ 40 = 1)	1.420(0.891–2.262)	0.140			0.927(0.656–1.308)	0.665		
ALT, U/l (≤ 40 = 1)	1.033(0.584–1.830)	0.910			0.795(0.526–1.203)	0.278		
AP, U/L(≤100 = 1)	2.058(0.648–6.542)	0.221			1.959(0.800–4.800)	0.141		
CRP, mg/l (≤8.2 = 1)	1.184(0.745–1.883)	0.475			1.702(1.169–2.478)	0.006	**1.791(1.122–2.858)**	**0.015**
GGT, U/l (≤ 100 = 1)	0.927(0.425–2.021)	0.848			1.448(0.707–2.966)	0.312		
Hemoglobin, g/dl (≤ 13 = 1)	0.585(0.354–0.965)	0.036	0.790(0.439–1.421)	0.431	0.760(0.525–1.100)	0.146		
INR (≤ 1 = 1)	1.283(0.816–2.016)	0.281			1.311(0.924–1.861)	0.130		
Platelet count, L/nl (≤ 300 = 1)	1.342(0.862–2.089)	0.192			0.895(0.633–1.264)	0.527		
Prothrombin time (≤ 110 = 1)	0.937(0.541–1.625)	0.818			0.674(0.419–1.086)	0.105		
Bilirubin, mg/dl (≤ 1 = 1)	1.308(0.839–2.041)	0.236			1.189(0.844–1.673)	0.322		
Intraoperative PRBC (No =1)	2.254(1.441–3.526)	<0.001	1.543(0.895–2.660)	0.119	2.308(1.624–3.279)	<0.001	1.226(0.768–1.957)	0.394
Intraoperative FFP (No =1)	2.219(1.402–3.512)	0.001	**2.759(1.688–4.508)**	**<0.001**	2.254(1.572–3.232)	<0.001	**1.742(1.089–2.787)**	**0.021**
Operative time, min (≤ 360 = 1)	1.869(1.023–3.415)	0.042	1.500(0.770–2.924)	0.233	1.605(1.031–2.497)	0.036	1.415(0.834–2.401)	0.198
LVI (No =1)	2.816(1.732–4.579)	<0.001	**2.193(1.252–3.840)**	**0.006**	2.034(1.380–3.000)	<0.001	**1.763(1.042–2.983)**	**0.035**
MVI (No =1)	1.012(0.993–1.031)	0.214			1.012(0.998–1.026)	0.084		
R1 resection (R0/Rx = 1)	2.478(1.487–4.128)	<0.001	1.204(0.655–2.215)	0.550	2.008(1.335–3.018)	0.001	1.763(1.042–2.983)	0.370
pT category (T1/T2 = 1)	2.424(1.549–3.793)	<0.001	**1.864(1.159–2.999)**	**0.010**	2.004(1.416–2.838)	<0.001	**1.661(1.068–2.584)**	**0.024**
pN category(N0 = 1)	2.427(1.552–3.793)	<0.001	**1.831(1.087–3.086)**	**0.023**	1.692(1.198–2.388)	0.003	1.127(0.691–1.839)	0.632
Tumor grading (G1/G2 = 1)	2.596(1.603–4.203)	<0.001	**1.911(1.138–3.208)**	**0.014**	2.164(1.487–3.150)	<0.001	1.340(0.845–2.123)	0.213
ICU time, days (≤ 1 = 1)	1.467(0.932–2.308)	0.098			2.074(1.464–2.938)	<0.001	1.286(0.820–2.017)	0.274
Hospitalization, days (≤ 14 = 1)	1.691(1.052–2.719)	0.030	1.012(0.573–1.787)	0.968	1.459(1.022–2.084)	0.037	1.000(0.999–1.001)	0.995
Perioperative complications (Clavien‐Dindo 0/I/II/IIIa =1)	1.382(0.858–2.227)	0.183			3.070(2.172–4.340)	<0.001	**2.476(1.422–4.310)**	**0.001**
Liver failure (No = 1)	1.545(0.809–2.951)	0.187			2.970(1.967–4.484)	<0.001	**1.961(1.139–3.375)**	**0.015**
Bile leak (No = 1)	2.714(1.695–4.344)	<0.001	**2.538(1.512–4.257)**	**<0.001**	1.823(1.252–2.656)	0.002	1.539(0.970–2.442)	0.067
Hemorrhage (No = 1)	1.520(0.865–2.671)	0.146			1.860(1.242–2.787)	0.003	1.573(0.784–2.041)	0.335
Infection Clavien‐Dindo (Clavien‐Dindo 0/I/II/IIIa =1)	2.101(1.145–3.857)	0.017	1.097(0.626–1.922)	0.746	3.773(2.567–5.547)	<0.001	1.391(0.758–2.550)	0.287
Adjuvant therapy (No = 1)	0.996(0.925–1.074)	0.924			1.013(0.992–1.035)	0.229		
BMI, kg/m^2^ (≤ 25 = 1)	1.398(0.895–2.185)	0.141			1.225(0.871–1.724)	0.243		
Visceral fat area, cm^2^(<100 = 1)	0.996(0.622–1.596)	0.987			1.186(0.816–1.724)	0.371		
Sarcopenia (No = 1)	1.395(0.888–2.194)	0.149			1.410(0.995–2.000)	0.054		
Myosteatosis (No = 1)	0.770(0.492–1.206)	0.253			1.214(0.864–1.705)	0.264		
Sarcopenic obesity (No = 1)	1.027(0.600–1.760)	0.922			1.155(0.775–1.721)	0.478		
VSR (Low VSR = 1)	0.948(0.583–1.542)	0.829			0.985(0.678–1.433)	0.939		

*Note*: Variables displaying a *p* < 0.05 in the univariate Cox Regression were transferred into a multivariable Cox regression model. Bold values display significance (*p* < 0.05).

Abbreviations: ALT, alanine aminotransferase; ASA, American Society of Anesthesiologists; AST, aspartate aminotransferase; BMI, body mass index; CRP, Creactive protein; FFP, fresh frozen plasma; GGT, gamma‐glutamyl transferase; HR, hazard ratio; iCCA, intrahepatic cholangiocarcinoma; ICU, intensive care unit; INR, international normalized ratio; LVI, lymph vascular invasion; MVI, microvascular invasion; PRBC, packed red Blood Cells; PVE, portal vein embolization; VSR, visceral to subcutaneous adipose tissue ratio.

Regarding OS, the univariate analysis revealed significant correlations with ASA score (*p* = 0.033), cholangitis (*p* = 0.017), CRP level (*p* = 0.006), intraoperative PRBC transfusion (*p* < 0.001), intraoperative FFP transfusion (*p* < 0.001), operative time (*p* = 0.036), LVI (*p* < 0.001), R1 resection status (*p* = 0.001), T category (*p* < 0.001), N category (*p* = 0.003), tumor grade (*p* < 0.001), duration of ICU stay (*p* < 0.001), duration of hospitalization (*p* = 0.037), perioperative complications (*p* < 0.001), liver failure (*p* < 0.001), bile leak (*p* = 0.002), postoperative hemorrhage (*p* = 0.003), and infectious complications (*p* < 0.001). In the multivariate Cox regression model, the independent predictors of OS were identified as ASA score (HR = 2.033, *p* = 0.003), CRP level (HR = 1.791, *p* = 0.015), intraoperative FFP transfusion (HR = 1.742, *p* = 0.021), LVI (HR = 1.763, *p* = 0.035), T category (HR = 1.661, *p* = 0.024), postoperative complications (HR = 2.476, *p* = 0.001), and liver failure (HR = 1.961, *p* = 0.015) (Table 3).

## 4 DISCUSSION

In this study, we aimed to assess the association between preoperative BC and postoperative complications in pCCA patients undergoing curative‐intent surgery. Severe malnutrition and cachexia, commonly observed in individuals with cancer, detrimentally impact the prognosis and can increases the likelihood of infections and mortality in this patient population.^19^ Additionally, it may contribute to a higher occurrence of cancer‐related complications.^20^ Despite notable advancements in intraoperative and perioperative care in recent years, postoperative complications continue to significantly impact patients short‐ and long‐term quality of life following surgery.^5^ Thus, effectively reducing the occurrence of postoperative complications and enhancing the curative outcomes for pCCA have emerged as an area of significant clinical concern.

In our study, we observed a notable proportion of patients diagnosed with pCCA to exhibit considerable alterations in BC. Specifically, 52.5% of the study set displayed obesity, 55.9% sarcopenia, 48.5% myosteatosis, 69.1% visceral obesity, 21.6% sarcopenic obesity and 32.4% high VSR. Regarding postoperative complications, a substantial association was shown between obesity, sarcopenia, myosteatosis and the actual occurrence of severe postoperative complications (Clavien‐Dindo≥3b). As severe postoperative complications were also associated with impaired OS, the clinical importance of altered BC is underlined by our data. Furthermore, Kaplan–Meier analysis showed a correlation between sarcopenia and OS in pCCA patients. Crucially, our findings illuminate the potential influence of BC on both postoperative complications and long‐term survival in pCCA, providing a novel contribution to the field. This study does further constitute the first comprehensive analysis of various measures of BC in the context of complications after surgery for pCCA and the first evidence that myosteatosis is a major predictor in postoperative outcome in these patients.

The occurrence of postoperative complications significantly impacts the prognosis following CCA surgery.^21^ Therefore, mitigating risk factors associated with these complications might help to enhance the prognosis of patients with CCA. Recent studies have identified several risk factors linked to postoperative complications in this patient population. Yuichi et al. demonstrated that patients with low psoas muscle index (PMI) or psoas muscle density (PMD) were more prone to infectious complications after surgery, particularly among those with pCCA.^8^ Kumamoto et al. discovered that BMI and neutrophil to lymphocyte ratio (NLR) were significant predictors of postoperative complications.^22^ Other reports have indicated that preoperative 6‐min walk distance are associated with postoperative complications in individuals with CAA.^23^

A comprehensive retrospective study indicates a potential correlation between obesity and both surgical wound infections and extended operative duration.^24^ Our findings validate the notion that significant postoperative complications in pCCA patients are linked to BMI ≥25 kg/m^2^, which aligns with previous research demonstrating a comparable impact of high BMI on postoperative complications in distal CCA treated by pancreatic resections.^22^ This is also consistent with our previous observations and findings.^10^ Impaired immune function may also contribute to the risk of severe complications in obese individuals.^25^ Furthermore, obesity is associated with reduced therapeutic tissue levels of applied antibiotics which can play a role in the effective management of complications.^26^

Sarcopenia, characterized by progressive muscle mass and function loss, is commonly observed in the elderly patients with malignancies.^27^ It can result from an imbalance between insufficient food intake and high tumor metabolism, leading to a negative energy balance.^27^ Patients with pCCA are particularly susceptible to obstructive jaundice, which can also contribute to inadequate intake and reduced physical activity, thereby inducing sarcopenia.^28^ Sarcopenia has been identified as a significant risk factor for poor perioperative outcomes undergoing hepatectomy within 90 days in previous studies, further supporting our findings as we have observed a strong association between sarcopenia and severe postoperative complications.^29^ Interestingly, previous studies have also shown that sarcopenia does not appear to be associated with postoperative complications in patients with pCCA, likely due to the limited sample size of the previous studies.^7^, ^10^

Myosteatosis is a pathological condition characterized by the accumulation of fat within muscle tissue, leading to impaired muscle and mitochondrial function.^30^ A meta‐analysis has revealed that myosteatosis as a significant predictor of survival in various types of cancer, with a 73% higher mortality rate reported in cancer patients with myosteatosis compared to those without.^31^ Our study provides novel evidence suggesting that myosteatosis is also an important predictor of serious complications following surgery for pCCA. This association may be explained by the inhibitory effects of myosteatosis on glutamine synthetase and its role in increasing inflammatory cytokines, ultimately impairing ammonia detoxification, a critical process for maintaining physiological balance.^32^

Lymph node metastasis represents the predominant form of metastasis in pCCA, thus making lymph node dissection a crucial component of pCCA surgery. Notably, lymph node metastasis in patients with pCCA significantly influences rates of early postoperative recurrence.^33^ Patients without lymph node metastasis exhibit a superior overall survival compared to those with positive lymph nodes.^34^ Moreover, nodal status correlates with disease‐specific survival.^35^ Interestingly, our study indicates a higher likelihood of lymph node metastasis in obese patients, while patients with sarcopenic obesity show a reduced likelihood. Further exploration is required to understand the underlying reasons for this observation. Additionally, it is worth noting that the median survival in patients with lymph node metastases does not differ between those who underwent complete (R0) or incomplete (R1) resection.^36^

In patients diagnosed with pCCA, liver function is commonly compromised due to preoperative cholestasis and cholangitis. In a study conducted by Ribero et al., which included a cohort of 133 pCCA patients, the occurrence of preoperative cholangitis was identified as a significant risk factor for postoperative hepatic failure and mortality following hepatectomy.^37^ Subsequent studies have reported similar findings, linking preoperative cholangitis in pCCA patients to increased postoperative mortality.^38^ However, the precise underlying pathophysiological mechanisms remain unknown. In our study, an association between the presence of preoperative cholangitis and myosteatosis was observed. Based on these findings, we hypothesize that preoperative cholangitis might be one of the factors influencing the development of myosteatosis and thereby, subsequently impacting the prognosis of patients with pCCA.

Furthermore, sarcopenia was found to have a negative impact on OS, but no significant correlation with RFS was observed, consistent with earlier research.^29^ Both sarcopenia and myosteatosis represent a loss of muscle function and quality. Inflammatory responses and oxidative stress are primary etiological factors in muscle wasting.^39^ Inflammatory cytokines can disrupt the balance between muscle protein synthesis and degradation, leading to muscle wasting,^40^ including chronic inflammation driven by interleukin‐1, interleukin‐6, and tumor necrosis factor‐alpha, which are associated with sarcopenia.^41^ Sarcopenia as one of the features of poor prognosis in cancer may be attributed to systemic inflammation and cachexia induced by the malignancy. An intriguing role for muscle tissue is to modulate immune regulation through the secretion of peptides and cytokines.^42^ The maintenance of immune function is further supported by muscle production of the cytokines interleukin‐7 (IL‐7) and interleukin‐15 (IL‐15).^43^ Consequently, we postulate that the systemic inflammatory response and alterations in immune function triggered by myosteatosis and sarcopenia are associated with postoperative complications and OS in patients with pCCA. In a subgroup analysis, we also found that patients with concurrent myosteatosis and sarcopenia similarly affect OS. We hypothesize that the concurrent presence of myosteatosis and sarcopenia may exert a compounded effect on OS.

Our study has shown that BC significantly influences the occurrence of postoperative complications in pCCA, which in turn impacts the prognosis of patients with pCCA. Previous research has underscored the vital role of muscle health in improving the prognosis of surgical patients, emphasizing the need for a comprehensive assessment of patients' nutritional status and muscle composition to enhance surgical outcomes.^44^ Therefore, improving BC becomes also crucial for pCCA patients. As such, Raff et al. discussed a prehabilitation program designed for cancer patients undergoing neoadjuvant treatment, aimed at improving patients' physical, nutritional, and psychological status before surgery.^45^ Some studies suggest that a combination of physical activity and nutritional supplementation promotion may be an effective intervention for patients with sarcopenia, which is often associated with pCCA.^46^ The engagement in physical exercise also contributes to the enhancement of skeletal muscle strength and quality and attenuate inflammatory responses, potentially preventing complications.^47^, ^48^ Perioperative nutritional supplementation has the potential to reduce the risk of postoperative complications and shorten the length of hospital stay for patients.^49^ In a study involving patients undergoing surgery for pancreatic cancer, physical functioning showed improvement by a home‐based rehabilitation program implemented both before and after the surgery.^50^ Leucine is known to directly initiate the translation of mRNA and plays a critical role in the regulation of muscle protein synthesis in both humans and rodents.^51^ Given the pivotal role of leucine in protein synthesis, supplementation with this amino acid may be beneficial in enhancing muscle protein synthesis. Previous studies have shown that a short‐term preoperative exercise program combined with the consumption of a leucine‐enriched beverage may contribute to increases in body weight, skeletal muscle mass, and SMI, thereby improving patient prognosis.^52^ However, there is currently a lack of research on strategies to improve BC specifically in patients with CCA. Further experiments and studies are needed to address this gap and analyze potential interventions in the future.

There are several limitations to this study. It is important to note that this was a retrospective study conducted at a single hepatobiliary center. Although the sample size is sufficient, further validation in multiple institutions or countries is necessary to enhance the generalizability of the findings. Further, the impact of rehabilitation on the preoperative or postoperative improvement of BC parameters in patients with pCCA remains unclear. Thus, the relationship between these improvements and their potential effects on reducing postoperative complications and long‐term survival needs further investigation. Addressing these clinical questions will require multi‐institutional randomized controlled studies in the future. Despite our efforts to control for multiple potential confounding factors by multivariable analysis, as with all observational studies, there may still be variables that are either unidentified or not adequately adjusted. Further, it must be noted that we exclusively addressed patients who underwent surgical resection for pCCA. Thus, our data cannot be used to draw conclusions for the wider population of pCCA patients who are not surgical candidates due to local irresectability or distant metastases.

Future studies should also emphasize the phenomenon of changes of BC over time both preoperatively as well as in the postoperative setting during oncological follow‐up. Changes of BC over time might even give more information about the individual patient's prognosis and susceptibility to postoperative complications. Unfortunately, our retrospective data set does not allow a longitudinal assessment in the study patients. This should be addressed in future prospective clinical cohorts.

## 5 CONCLUSION

Our study indicates that changes in BC are strongly linked to postoperative complications in pCCA patients. Consequently, assessing BC prior to surgery in pCCA patients could serve as a valuable tool for risk perioperative stratification. In the future, it is essential to focus on monitoring BC changes before surgery in pCCA cases and develop suitable nutritional and exercise programs to enhance BC prior to the actual surgical procedure. By addressing BC status preoperatively, we may potentially optimize patient outcomes and minimize the risk of postoperative complications. To achieve this goal, further research and the development of tailored interventions are needed to effectively improve BC in pCCA patients.

## AUTHOR CONTRIBUTIONS

**Guanwu Wang:** Conceptualization (equal); data curation (equal); funding acquisition (equal); investigation (equal); software (equal); writing – original draft (equal); writing – review and editing (equal). **Anna Mantas:** Writing – original draft (equal); writing – review and editing (equal). **Lara Heij:** Writing – original draft (equal); writing – review and editing (equal). **Tarick Al‐Masri:** Writing – original draft (equal); writing – review and editing (equal). **Dong Liu:** Writing – original draft (equal); writing – review and editing (equal). **Daniel Heise:** Writing – original draft (equal); writing – review and editing (equal). **Sophia Schmitz:** Writing – original draft (equal); writing – review and editing (equal). **Steven W. M. Olde Damink:** Writing – original draft (equal); writing – review and editing (equal). **Tom Luedde:** Writing – original draft (equal); writing – review and editing (equal). **Sven A. Lang:** Writing – original draft (equal); writing – review and editing (equal). **Tom F. Ulmer:** Writing – original draft (equal); writing – review and editing (equal). **Ulf P. Neumann:** Writing – original draft (equal); writing – review and editing (equal). **Jan Bednarsch:** Conceptualization (equal); data curation (equal); investigation (equal); methodology (equal); project administration (equal); software (equal); writing – original draft (equal); writing – review and editing (equal).

## FUNDING INFORMATION

Guanwu Wang was funded by China Scholarship Council (Grant number: 202108430018). Dong Liu was funded by China Scholarship Council (Grant number: 202208080011).

## CONFLICT OF INTEREST STATEMENT

The authors of this manuscript have no conflict of interest to declare.

## ETHICS STATEMENT

This study was conducted in accordance with the current version of the Declaration of Helsinki and good clinical practice guidelines (International Conference on Harmonization, Good Clinical Practice). Approval was granted by the institutional review board (EK 23‐269).

## Supporting information


Table S1.

## Data Availability

The data that support the findings of this study are available from the corresponding author upon reasonable request.
