# Supplementary material for: Body composition is associated with postoperative complications in perihilar cholangiocarcinoma
Source: Cancer Med. 2024 Jan 1;13(1):e6878. doi: 10.1002/cam4.6878 (PMC10807576; doi:10.1002/cam4.6878)
Supplement: Supplementary file 1 — Table S1. [file CAM4-13-e6878-s001.docx]

**Supplementary Table 1 Detailed univariate analysis and multivariate analysis of clinical and pathological data associated with body composition in perihilar cholangiocarcinoma**

| **Outcome** | **Desriptives** | | **Univariate analysis** | | **Multivariate Analysis** | | |
| --- | --- | --- | --- | --- | --- | --- | --- |
| **BMI** | **＜25(n=97）** | **≥25(n=107)** | **OR (95% CI)** | **p-value** | **OR (95% CI)** | | **p-value** |
| Sex  (male/female(%); ref=male) | 62(63.9)/35(36.1) | 74(69.2)/33(30.8) | 0.790(0.441-1.415) | 0.428 |  | |  |
| Age  (≤65/ >65 years(%); ref=≤65) | 39(40.2)/58(59.8) | 48(44.9)/59(55.1) | 0.827(0.474-1.442) | 0.502 |  | |  |
| ASA  ((I/II)/(III/IV) (%);ref= I/II) | 39(40.2)/58(59.8) | 38(35.5)/69(64.5) | 1.221(0.693-2.153) | 0.490 |  | |  |
| Cholangitis  (No/Yes(%);ref=No) | 68(70.1)/29(29.9) | 71(66.4)/36(33.6) | 1.189(0.658-2.148) | 0.566 |  | |  |
| PVE  (No/Yes(%);ref=No) | 66(68.0)/31(32.0) | 67(62.6)/40(37.4) | 1.271(0.712-2.268) | 0.417 |  | |  |
| Neoadjuvant therapy  ((No/Yes(%);ref=No) | 92(94.8)/5(5.2) | 102(95.3)/5(4.7) | 0.902(0.253-3.216) | 0.874 |  | |  |
| AST, U/L  (≤40/ >40(%); ref=≤40) | 36(37.1)/60(61.9) | 50(46.7)/54(50.5) | 0.648(0.368-1.140) | 0.132 |  | |  |
| ALT, U/L  (≤40/ >40(%); ref=≤40) | 19(19.6)/59(60.8) | 33(30.8)/58(54.2) | 0.566(0.289-1.107) | 0.096 |  | |  |
| AP,U/L  (≤100/ >100(%); ref=≤100) | 2(2.1)/91(93.8) | 10(9.3)/94(87.9) | 0.207(0.044-0.969) | 0.045 | **0.081(0.010-0.660)** | | **0.019** |
| CRP,mg/l  (≤8.2/ >8.2(%); ref=≤8.2) | 41(42.3)/55(56.7) | 43(40.2)/62(57.9) | 1.254(0.704-2.235) | 0.442 |  | |  |
| GGT,U/L  (≤100/ >100(%); ref=≤100) | 5(5.2)/88(90.7) | 11(10.3)/93(86.9) | 0.480(0.160-1.438) | 0.190 |  | |  |
| Hemoglobin, g/L  (≤13/ >13(%); ref=≤13) | 71(73.2)/25(25.8) | 67(62.6)/40(37.4) | 1.696(0.930-3.092) | 0.085 |  | |  |
| INR  (≤1/>1(%); ref=≤1) | 38(39.2)/57(58.8) | 41(38.3)/66(61.7) | 1.073(0.609-1.890) | 0.807 |  | |  |
| Platelet count  (≤250/ >250(%); ref=≤250) | 47(48.5)/49(50.5) | 63(58.9)/44(41.1) | 0.670(0.384-1.167) | 0.157 |  | |  |
| Prothrombin time  (≤110/ >110(%); ref=≤110) | 81(83.5)/14(14.4) | 87(81.3)/20(18.7) | 1.330(0.630-2.870) | 0.454 |  | |  |
| Total bilirubin,mg/dl  (≤1/ >1(%); ref=≤1) | 37(38.1)/60(61.9) | 55(51.4)/52(48.6) | 0.583(0.334-1.019) | 0.058 |  | |  |
| Intraop PRBC  (No/Yes(%);ref=No) | 46(47.4)/51(52.6) | 57(53.3)/50(46.7) | 0.791(0.456-1.372) | 0.404 |  | |  |
| Intraop FFP  ((No/Yes(%);ref=No) | 45(46.4)/52(53.6) | 50(46.7)/57(53.3) | 0.987(0.569-1.711) | 0.962 |  | |  |
| Operative time ,minutes  (≤360/ >360(%); ref=≤360) | 20(20.6)/77(79.4) | 19(17.8)/88(82.2) | 1.203(0.589-2.419) | 0.604 |  | |  |
| Time to surgery,days  (≤30/ >30(%); ref=≤30) | 32(33.0)/53(54.6) | 28(26.2)/64(59.8) | 1.380(0.739-2.576) | 0.312 |  | |  |
| LVI  (No/Yes(%);ref=No) | 71(73.2)/20(20.6) | 79(73.8)/25(23.4) | 1.123(0.575-2.195) | 0.733 |  | |  |
| MVI  (No/Yes(%);ref=No) | 65(67.0)/28(28.9) | 70(65.4)/34(31.8) | 0.999(0.971-1.028) | 0.952 |  | |  |
| R1 resection  ((R0/R2)/R1) (%); ref=R0/R2) | 75(77.3)/20(20.6) | 87(81.3)/20(18.7) | 0.862(0.431-1.723) | 0.674 |  | |  |
| pT category  (T1-2/T3-4(%);ref=T1-T2) | 61(62.9)/34(35.1) | 72(67.3)/35(32.7) | 0.872(0.487-1.561) | 0.645 |  | |  |
| pN category  (N0/N1(%);ref=N0) | 47(48.5)/49(50.5) | 68(63.6)/39(36.4) | 0.550(0.314-0.964) | 0.037 | **0.507(0.279-0.920)** | | **0.025** |
| Tumor grading  ((G1/G2)/( G3/G4) (%);ref= G1/G2) | 66(68.0)/26(26.8) | 77(72.0)/27(25.2) | 0.890(0.474-1.673) | 0.718 |  | |  |
| ICU time,days  (≤1/>1(%),ref=1） | 53(54.6)/44(45.4) | 59(55.1)/48(44.9) | 0.980(0.564-1.702) | 0.943 |  | |  |
| Hospitalization,days  (≤14/>14(%); ref=≤14) | 44(45.4)/53(54.6) | 40(37.4)/67(62.6) | 1.391(0.795-2.433) | 0.248 |  | |  |
| Postoperative complications, Clavien-Dindo ((0/I/II/IIIa)/(IIIa/IV/V) (%);ref= 0/I/II/IIIa) | 64(66.0)/33(34.0) | 56(52.3)/51(47.7) | 1.766(1.003-3.111) | 0.049 | **2.001(1.097-3.653)** | | **0.024** |
| Liver failure  (No/Yes(%);ref=No) | 75(77.3)/22(22.7) | 92(86.0)/15(14.0) | 0.556(0.270-1.146) | 0.112 |  | |  |
| Bile leak  (No/Yes(%);ref=No) | 76(78.4)/21(21.6) | 79(73.8)/28(26.2) | 1.283(0.671-2.451) | 0.451 |  | |  |
| Hemorrhage  (No/Yes(%);ref=No) | 79(81.4)/18(18.6) | 86(80.4)/21(19.6) | 1.072(0.532-2.158) | 0.846 |  | |  |
| Infection Clavien-Dindo  ((0/I/II/IIIa)/(IIIa/IV/V) (%);ref= 0/I/II/IIIa) | 77(79.4)/19(19.6) | 79(73.8)/28(26.2) | 1.436(0.741-2.784) | 0.283 |  | |  |
| Adjuvant therapy  (No/Yes(%);ref=No) | 66(68.0)/26(26.8) | 73(68.2)/28(26.2) | 0.943(0.693-1.282) | 0.706 |  | |  |
| **Sarcopenia** | **No****(n=90)** | **Yes(n=114)** | **OR (95% CI)** | **p-value** | **OR (95% CI)** | | **p-value** |
| Sex  (male/female(%); ref=male) | 78(86.7)/12(13.3) | 58(50.9)/56(49.1) | 6.276(3.085-12.766) | ＜0.001 | 8.253(3.600-18.917) | | ＜0.001 |
| Age  (≤65/ >65 years(%); ref=≤65) | 41(45.6)/49(54.4) | 46(40.4)/68(59.6) | 1.237(0.707-2.163) | 0.456 |  | |  |
| ASA  ((I/II)/(III/IV) (%);ref= I/II) | 30(33.3)/60(66.7) | 47(41.2)/67(58.8) | 0.713(0.401-1.267) | 0.249 |  | |  |
| Cholangitis  (No/Yes(%);ref=No) | 62(68.9)/28(31.1) | 77(67.5)/37(32.5) | 1.064(0.587-1.927) | 0.838 |  | |  |
| PVE  (No/Yes(%);ref=No) | 58(64.4)/32(35.6) | 75(65.8)/39(34.2) | 0.943(0.528-1.683) | 0.841 |  | |  |
| Neoadjuvant therapy  ((No/Yes(%);ref=No) | 85(94.4)/5(5.6) | 109(95.6)/5(4.4) | 0.780(0.219-2.781) | 0.701 |  | |  |
| AST, U/L  (≤40/ >40(%); ref=≤40) | 36(40.0)/51(56.7) | 50(43.9)/63(55.3) | 0.889(0.505-1.566) | 0.685 |  | |  |
| ALT, U/L  (≤40/ >40(%); ref=≤40) | 16(17.8)/58(64.4) | 36(31.6)/59(51.8) | 0.452(0.226-0.903) | 0.024 | 0.536(0.270-1.273) | | 0.109 |
| AP,U/L  (≤100/ >100(%); ref=≤100) | 3(3.3)/86(95.6) | 9(7.9)/99(86.8) | 0.384(0.101-1.463) | 0.161 |  | |  |
| CRP,mg/l  (≤8.2/ >8.2(%); ref=≤8.2) | 38(42.2)/52(57.8) | 46(40.4)/65(57.0) | 0.897(0.502-1.604) | 0.714 |  | |  |
| GGT,U/L  (≤100/ >100(%); ref=≤100) | 6(6.7)/83(92.2) | 10(8.8)/98(86.0) | 0.708(0.247-2.032) | 0.521 |  | |  |
| Hemoglobin, g/L  (≤13/ >13(%); ref=≤13) | 57(63.3)/33(36.7) | 81(71.1)/32(28.1) | 0.682(0.377-1.234) | 0.206 |  | |  |
| INR  (≤1/>1(%); ref=≤1) | 35(38.9)/54(60.0) | 44(38.6)/69(60.5) | 1.016(0.575-1.796) | 0.955 |  | |  |
| Platelet count  (≤300/ >300(%); ref=≤250) | 53(58.9)/37(41.1) | 57(50.0)/56(49.1) | 1.299(0.726-2.323) | 0.379 |  | |  |
| Prothrombin time  (≤110/ >110(%); ref=≤110) | 76(84.4)/13(14.4) | 92(80.7)/21(18.4) | 1.334(0.627-2.841) | 0.454 |  | |  |
| Total bilirubin,mg/dl  (≤1/ >1(%); ref=≤1) | 37(41.1)/53(58.9) | 55(48.2)/59(51.8) | 0.749(0.429-1.308) | 0.310 |  | |  |
| Intraop PRBC  (No/Yes(%);ref=No) | 46(51.1)/44(48.9) | 57(50.0)/57(50.0) | 1.045(0.601-1.817) | 0.875 |  | |  |
| Intraop FFP  ((No/Yes(%);ref=No) | 46(51.1)/44(48.9) | 49(43.0)/65(57.0) | 1.387(0.796-2.416) | 0.248 |  | |  |
| Operative time minutes  (≤360/ >360(%); ref=≤360) | 21(23.3)/69(76.7) | 18(15.8)/96(84.2) | 1.623(0.805-3.273) | 0.176 |  | |  |
| Time to surgery,days  (≤30/ >30(%); ref=≤30) | 23(25.6)/55(61.1) | 37(32.5)/62(54.4) | 0.701(0.372-1.322) | 0.272 |  | |  |
| LVI  (No/Yes(%);ref=No) | 70(77.8)/18(20.0) | 80(70.2)/27(23.7) | 1.312(0.667-2.583) | 0.431 |  | |  |
| MVI  (No/Yes(%);ref=No) | 61(67.8)/27(30.0) | 74(64.9)/35(30.7) | 0.998(0.970-1.027) | 0.888 |  | |  |
| R1 resection  ((R0/R2)/R1) (%); ref=R0/R2) | 71(78.9)/18(20.0) | 91(79.8)/22(19.3) | 0.954(0.475-1.913) | 0.894 |  | |  |
| pT category  (T1-2/T3-4(%);ref=T1-T2) | 57(63.3)/32(35.6) | 76(66.7)/37(32.5) | 0.867(0.483-1.556) | 0.633 |  | |  |
| pN category  (N0/N1(%);ref=N0) | 48(53.3)/42(46.7) | 67(58.8)/46(40.4) | 0.785(0.449-1.372) | 0.395 |  | |  |
| Tumor grading  ((G1/G2)/( G3/G4) (%);ref= G1/G2) | 59(65.6)/27(30.0) | 84(73.7)/26(22.8) | 0.676(0.359-1.274) | 0.226 |  | |  |
| ICU time  days(≤1/>1(%),ref=1） | 55(61.1)/35(38.9) | 57(50.0)/57(50.0) | 1.571(0.897-2.753) | 0.114 |  | |  |
| Hospitalization  days(≤14/>14(%); ref=≤14) | 60(66.7)/30(33.3) | 60(52.6)/54(47.4) | 1.080(0.616-1.894) | 0.787 |  | |  |
| Postoperative complications Clavien-Dindo  ((0/I/II/IIIa)/(IIIa/IV/V) (%);ref= 0/I/II/IIIa) | 37(41.1)/29(32.2) | 37(32.5)/44(38.6) | 1.800(1.016-3.189) | 0.044 | **2.145(1.057-4.350)** | | **0.034** |
| Liver failure  (No/Yes(%);ref=No) | 75(83.3)/15(16.7) | 92(80.7)/22(19.3) | 1.196(0.580-2.465) | 0.628 |  | |  |
| Bile leak  (No/Yes(%);ref=No) | 71(78.9)/19(21.1) | 84(73.7)/30(26.3) | 1.355(0.693-2.571) | 0.388 |  | |  |
| Hemorrhage  (No/Yes(%);ref=No) | 73(81.1)/17(18.) | 92(80.7)/22(19.3) | 1.027(0.508-2.075) | 0.941 |  | |  |
| Infection Clavien-Dindo  ((0/I/II/IIIa)/(IIIb/IV/V) (%);ref= 0/I/II/IIIa) | 74(82.2)/15(16.7) | 82(71.9)/32(28.1) | 1.925(0.966-3.835) | 0.062 |  | |  |
| Adjuvant therapy  (No/Yes(%);ref=No) | 62(68.9)/23(25.6) | 77(67.5)/31(27.2) | 0.954(0.803-1.134) | 0.595 |  | |  |
| **Myosteatosis** | **No(n=105)** | **Yes(n=99)** | **OR (95% CI)** | **p-value** | **OR (95% CI)** | | **p-value** |
| Sex  (male/female(%); ref=male) | 78(76.3)/27(25.7) | 58(58.6)/41(41.4) | 2.042(1.129-3.694) | 0.018 | **2.254(1.197-4.242)** | | **0.012** |
| Age  (≤65/ >65 years(%); ref=≤65) | 56(53.3)/49(46.7) | 31(31.3)/68(68.7) | 2.507(1.415-4.443) | 0.002 | **2.691(1.473-4.916)** | | **0.001** |
| ASA  ((I/II)/(III/IV) (%);ref= I/II) | 45(42.9)/60(57.1) | 32(32.3)/67(67.7) | 1.570(0.887-2.781) | 0.122 |  | |  |
| Cholangitis  (No/Yes(%);ref=No) | 79(75.2)/26(24.8) | 60(60.6)/39(39.4) | 1.975(1.085-3.595) | 0.026 | **1.965(1.039-3.719)** | | **0.038** |
| PVE  (No/Yes(%);ref=No) | 65(61.9)/40(38.1) | 68(68.7)/31(31.3) | 0.741(0.415-1.322) | 0.310 |  | |  |
| Neoadjuvant therapy((No/Yes(%);ref=No) | 99(94.3)/6(5.7) | 95(96.0)/4(4.0) | 0.695(0.190-2.539) | 0.582 |  | |  |
| AST, U/L  (≤40/ >40(%); ref=≤40) | 41(39.0)/61(58.1) | 45(45.5)/53(53.5) | 0.792(0.452-1.387) | 0.414 |  | |  |
| ALT, U/L  (≤40/ >40(%); ref=≤40) | 20(19.0)/64(61.0) | 32(32.3)/53(53.5) | 0.518(0.266-1.008) | 0.053 |  | |  |
| AP,U/L  (≤100/ >100(%); ref=≤100) | 5(4.8)/96(91.4) | 7(7.1)/89(89.9) | 0.662(0.203-2.162) | 0.495 |  | |  |
| CRP,mg/l  (≤8.2/ >8.2(%); ref=≤8.2) | 48(45.7)/54(51.4) | 36(36.4)/63(63.6) | 1.474(0.825-2.635) | 0.190 |  | |  |
| GGT,U/L  (≤100/ >100(%); ref=≤100) | 8(7.6)/93(88.6) | 8(8.1)/88(88.9) | 0.946(0.340-2.630) | 0.916 |  | |  |
| Hemoglobin, g/L  (≤13/ >13(%); ref=≤13) | 65(61.9)/40(38.1) | 73(73.7)/25(25.3) | 0.557(0.305-1.015) | 0.056 |  | |  |
| INR  (≤1/>1(%); ref=≤1) | 46(43.8)/57(54.3) | 33(33.3)/66(66.7) | 1.614(0.912-2.856) | 0.100 |  | |  |
| Platelet count  (≤300/ >300(%); ref=≤300) | 60(57.1)/45(42.9) | 50(50.5)/48(48.5) | 1.280(0.736-2.226) | 0.382 |  | |  |
| Prothrombin time  (≤110/ >110(%); ref=≤110) | 83(79.0)/20(19.0) | 85(85.9)/14(14.1) | 0.684(0.324-1.443) | 0.318 |  | |  |
| Total bilirubin,mg/dl  (≤1/ >1(%); ref=≤1) | 41(39.0)/64(61.0) | 51(51.5)/48(48.5) | 0.603(0.346-1.051) | 0.074 |  | |  |
| Intraop PRBC  (No/Yes(%);ref=No) | 57(54.3)/48(45.7) | 46(46.5)/53(53.5) | 1.368(0.789-2.373) | 0.265 |  | |  |
| Intraop FFP  ((No/Yes(%);ref=No) | 51(48.6)/54(51.4) | 44(44.4)/55(55.6) | 1.181(0.680-2.048) | 0.555 |  | |  |
| Operative time,minutes  (≤360/ >360(%); ref=≤360) | 17(16.2)/88(83.8) | 22(22.2)/77(77.8) | 0.676(0.335-1.366) | 0.275 |  | |  |
| Time to surgery,days  (≤30/ >30(%); ref=≤30) | 29(72.6)/60(57.1) | 31(31.3)/57(57.6) | 0.889 (0.477-1.657) | 0.710 |  | |  |
| LVI  (No/Yes(%);ref=No) | 79(75.2)/22(21.0) | 71(71.7)/23(23.2) | 1.163(0.597-2.266) | 0.657 |  | |  |
| MVI  (No/Yes(%);ref=No) | 67(63.8)/35(33.3) | 68(68.7)/27(27.3) | 1.043(0.937-1.161) | 0.443 |  | |  |
| R1 resection  ((R0/R2)/R1) (%); ref=R0/R2) | 87(82.9)/17(16.2) | 75(75.8)/23(23.2) | 1.569(0.780-3.157) | 0.206 |  | |  |
| pT category  (T1-2/T3-4(%);ref=T1-T2) | 69(65.7)/35(33.3) | 64(64.6)/34(34.3) | 1.047(0.585-1.874) | 0.876 |  | |  |
| pN category  (N0/N1(%);ref=N0) | 61(58.1)/43(41.0) | 54(54.5)/45(45.5) | 1.182(0.678-2.061) | 0.555 |  | |  |
| Tumor grading  ((G1/G2)/( G3/G4) (%);ref= G1/G2) | 70(66.7)/30(28.6) | 73(73.7)/23(23.2) | 0.735(0.390-1.387) | 0.342 |  | |  |
| ICU time,days(≤1/>1(%),ref=1） | 59(56.2)/46(43.8) | 53(53.5)/46(46.5) | 1.113(0.641-1.933) | 0.703 |  | |  |
| Hospitalization,days  (≤14/>14(%); ref=≤14) | 46(43.8)/59(56.2) | 38(38.4)/61(61.6) | 1.252(0.715-2.189) | 0.432 |  | |  |
| Postoperative complications Clavien-Dindo  ((0/I/II/IIIa)/(IIIa/IV/V) (%);ref= 0/I/II/IIIa) | 70(66.7)/35(33.3) | 50(50.5)/49(49.5) | 1.960(1.113-3.450) | 0.020 | **2.097(1.143-3.847)** | | **0.017** |
| Liver failure  (No/Yes(%);ref=No) | 87(82.9)/18(17.1) | 80(80.8)/19(19.2) | 1.148(0.563-2.341) | 0.704 |  | |  |
| Bile leak  (No/Yes(%);ref=No) | 80(76.2)/25(23.8) | 75(75.8)/24(24.2) | 1.024(0.539-1.947) | 0.942 |  | |  |
| Hemorrhage  (No/Yes(%);ref=No) | 89(84.8)/16(15.2) | 76(76.8)/23(23.2) | 1.683(0.830-3.416) | 0.149 |  | |  |
| Infection Clavien-Dindo  ((0/I/II/IIIa)/(IIIa/IV/V) (%);ref=0/I/II/IIIa) | 83(79.0)/21(20.0) | 73(73.7)/26(26.3) | 1.408(0.731-2.711) | 0.307 |  | |  |
| Adjuvant therapy  (No/Yes(%);ref=No) | 64(61.0)/35(33.3) | 75(75.8)/19(19.2) | 1.026(0.953-1.105) | 0.497 |  | |  |
| **Visceral obesity (cm^2^)** | **≤100(n=63)** | **＞100(n=141)** | **OR (95% CI)** | **p-value** | **OR (95% CI)** | | **p-value** |
| Sex  (male/female(%); ref=male) | 36(57.1)/39(38.2) | 100(70.9)/41(29.1) | 0.547(0.295-1.014) | 0.055 |  | |  |
| Age  (≤65/ >65 years(%); ref=≤65) | 31(49.2)/32(50.8) | 56(39.7)/85(60.3) | 1.470(0.808-2.674) | 0.206 |  | |  |
| ASA  ((I/II)/(III/IV) (%);ref= I/II) | 25(39.7)/38(60.3) | 52(36.9)/89(63.1) | 1.126(0.612-2.072) | 0.703 |  | |  |
| Cholangitis  (No/Yes(%);ref=No) | 46(73.0)/17(27.0) | 93(66.0)/48(34.0) | 1.397(0.724-2.692) | 0.319 |  | |  |
| PVE  (No/Yes(%);ref=No) | 41(65.1)/22(34.9) | 92(65.2)/49(34.8) | 0.993(0.532-1.851) | 0.981 |  | |  |
| Neoadjuvant therapy  ((No/Yes(%);ref=No) | 57(90.5)/6(9.5) | 137(97.2)/4(2.8) | 0.277(0.075-1.020) | 0.054 |  | |  |
| AST, U/L  (≤40/ >40(%); ref=≤40) | 26(41.3)/37(58.7) | 26(41.3)/37(58.7) | 0.902(0.493-1.651) | 0.738 |  | |  |
| ALT, U/L  (≤40/ >40(%); ref=≤40) | 16(25.4)/33(52.4) | 36(25.5)/84(59.6) | 1.131(0.554-2.309) | 0.735 |  | |  |
| AP,U/L  (≤100/ >100(%); ref=≤100) | 1(1.6)/60(95.2) | 11(7.8)/125(88.7) | 0.189(0.024-1.501) | 0.115 |  | |  |
| CRP,mg/l  (≤8.2/ >8.2(%); ref=≤8.2) | 27(42.9)/35(55.6) | 57(40.4)/82(58.2) | 1.197(0.645-2.224) | 0.569 |  | |  |
| GGT,U/L  (≤100/ >100(%); ref=≤100) | 3(4.8)/58(92.1) | 13(9.2)/123(87.2) | 0.489(0.134-1.784) | 0.279 |  | |  |
| Hemoglobin, g/L  (≤13/ >13(%); ref=≤13) | 45(71.4)/18(28.6) | 93(66.0)/47(33.3) | 1.263(0.660-2.419) | 0.480 |  | |  |
| INR  (≤1/>1(%); ref=≤1) | 27(42.9)/34(54.0) | 52(36.9)/89(63.1) | 1.359(0.738-2.502) | 0.324 |  | |  |
| Platelet count  (≤300/ >300(%); ref=≤300) | 31(49.2)/32(50.8) | 79(56.0)/61(43.3) | 0.748(0.412-1.358) | 0.340 |  | |  |
| Prothrombin time  (≤110/ >110(%); ref=≤110) | 51(81.0)/10(15.9) | 117(83.0)/24(17.0) | 1.046(0.466-2.346) | 0.913 |  | |  |
| Total bilirubin,mg/dl  (≤1/ >1(%); ref=≤1) | 26(41.3)/37(58.7) | 66(46.8)/75(53.2) | 0.799(0.438-1.456) | 0.463 |  | |  |
| Intraop PRBC  (No/Yes(%);ref=No) | 28(44.4)/35(55.6) | 75(53.2)/66(46.8) | 0.704(0.388-1.279) | 0.249 |  | |  |
| Intraop FFP  ((No/Yes(%);ref=No) | 28(44.4)/35(55.6) | 67(47.5)/74(52.5) | 0.884(0.486-1.605) | 0.684 |  | |  |
| Operative time minutes  (≤360/ >360(%); ref=≤360) | 13(20.6)/50(79.4) | 26(18.4)/115(81.6) | 1.150(0.547-2.420) | 0.713 |  | |  |
| Time to surgery,days  (≤30/ >30(%); ref=≤30) | 17(27.0)/40(63.5) | 43(30.5)/77(54.6) | 0.761(0.386-1.501) | 0.431 |  | |  |
| LVI  (No/Yes(%);ref=No) | 48(76.2)/13(20.6) | 102(72.3)/32(22.7) | 1.158(0.558-2.404) | 0.693 |  | |  |
| MVI  (No/Yes(%);ref=No) | 41(65.1)/20(31.7) | 94(66.7)/42(29.8) | 0.992(0.964-1.020) | 0.566 |  | |  |
| R1 resection  ((R0/R2)/R1) (%); ref=R0/R2) | 47(74.6)/15(23.8) | 99(70.2)/41(29.1) | 1.042(0.490-2.214) | 0.915 |  | |  |
| pT category  (T1-2/T3-4(%);ref=T1-T2) | 46(73.0)/16(25.4) | 87(61.7)/53(37.6) | 1.751(0.902-3.401) | 0.098 |  | |  |
| pN category  (N0/N1(%);ref=N0) | 34(54.0)/29(46.0) | 81(57.4)/59(41.8) | 0.854(0.469-1.553) | 0.605 |  | |  |
| Tumor grading  ((G1/G2)/( G3/G4) (%);ref= G1/G2) | 41(65.1)/19(30.2) | 102(72.3)/34(24.1) | 0.719(0.369-1.403) | 0.334 |  | |  |
| ICU time,days  (≤1/>1(%),ref=1） | 32(50.8)/31(49.2) | 80(56.7)/61(43.3) | 0.787(0.434-1.428) | 0.431 |  | |  |
| Hospitalization,days  (≤14/>14(%); ref=≤14) | 25(39.7)/38(60.3) | 59(41.8)/82(58.2) | 0.914(0.499-1.675) | 0.772 |  | |  |
| Postoperative complications Clavien-Dindo  ((0/I/II/IIIa)/(IIIa/IV/V) (%);ref= 0/I/II/IIIa) | 40(63.5)/23(36.5) | 80(56.7)/61(43.3) | 1.326(0.719-2.444) | 0.366 |  | |  |
| Liver failure  (No/Yes(%);ref=No) | 47(74.6)/16(25.4) | 120(85.1)/21(14.9) | 0.514(0.247-1.070) | 0.075 |  | |  |
| Bile leak  (No/Yes(%);ref=No) | 52(82.5)/11(17.5) | 103(73.0)/38(27.0) | 1.744(0.824-3.690) | 0.146 | |  |  |
| Hemorrhage  (No/Yes(%);ref=No) | 50(79.4)/13(20.6) | 115(81.6)/26(18.4) | 0.870(0.413-1.830) | 0.713 | |  |  |
| Infection Clavien-Dindo  ((0/I/II/IIIa)/(IIIa/IV/V) (%);ref=0/I/II/IIIa) | 48(76.2)/14(22.2) | 108(76.6)/33(23.4) | 1.048(0.514-2.134) | 0.898 |  | |  |
| Adjuvant therapy  (No/Yes(%);ref=No) | 42(66.7)/17(27.0) | 97(68.8)/37(26.2) | 0.928(0.608-1.416) | 0.727 |  | |  |
| **Sarcopenic obesity** | **No(n=160)** | **Yes(n=44)** | **OR (95% CI)** | **p-value** | **OR (95% CI)** | | **p-value** |
| Sex  (male/female(%); ref=male) | 100(62.5)/60(37.5) | 36(81.8)/8(18.2) | 0.370(0.161-0.850) | 0.019 | **0.170(0.056-0.519)** | | **0.002** |
| Age  (≤65/ >65 years(%); ref=≤65) | 73(45.6)/87(54.4) | 14(31.8)/30(68.2) | 1.798(0.887-3.645) | 0.104 |  | |  |
| ASA  ((I/II)/(III/IV) (%);ref= I/II) | 61(38.1)/99(61.9) | 16(36.4)/28(63.6) | 1.078(0.540-2.154) | 0.831 |  | |  |
| Cholangitis, (No/Yes(%);ref=No) | 113(70.6)/47(29.4) | 26(59.1)/18(40.9) | 1.664(0.834-3.320) | 0.148 |  | |  |
| PVE  (No/Yes(%);ref=No) | 106(66.3)/54(33.8) | 27(61.4)/17(38.6) | 1.236(0.620-2.463) | 0.547 |  | |  |
| Neoadjuvant therapy  ((No/Yes(%);ref=No) | 152(95.0)/8(5.0) | 42(95.5)/2(4.5) | 0.905(0.185-4.422) | 0.902 |  | |  |
| AST, U/L  (≤40/ >40(%); ref=≤40) | 61(38.1)/95(59.4) | 25(56.8)/19(43.2) | 0.488(0.248-0.961) | 0.038 | 0.550(0.192-1.576) | | 0.266 |
| ALT, U/L  (≤40/ >40(%); ref=≤40) | 34(21.3)/100(62.5) | 18(40.9)/17(38.6) | 0.321(0.149-0.693) | 0.004 | **0.248(0.102-0.604)** | | **0.002** |
| AP,U/L  (≤100/ >100(%); ref=≤100) | 8(5.0)/148(92.5) | 4(9.1)/37(84.1) | 0.500(0.143-1.751) | 0.278 |  | |  |
| CRP,mg/l  (≤8.2/ >8.2(%); ref=≤8.2) | 67(41.9)/91(56.9) | 17(38.6)/26(59.1) | 1.053(0.520-2.135) | 0.885 |  | |  |
| GGT,U/L  (≤100/ >100(%); ref=≤100) | 13(8.1)/143(89.4) | 3(6.8)/38(86.4) | 1.152(0.312-4.248) | 0.832 |  | |  |
| Hemoglobin, g/L  (≤13/ >13(%); ref=≤13) | 107(66.9)/52(32.5) | 31(70.5)/13(29.5) | 0.863(0.417-1.786) | 0.691 |  | |  |
| INR  (≤1/>1(%); ref=≤1) | 63(39.4)/95(59.4) | 16(36.4)/28(63.6) | 1.161(0.581-2.318) | 0.673 |  | |  |
| Platelet count  (≤300/ >300(%); ref=≤300) | 84(52.5)/75(46.9) | 26(59.1)/18(40.9) | 0.775(0.394-1.526) | 0.461 |  | |  |
| Prothrombin time  (≤110/ >110(%); ref=≤110) | 130(81.3)/28(17.5) | 38(86.4)/6(13.6) | 0.733(0.283-1.901) | 0.523 |  | |  |
| Total bilirubin,mg/dl  (≤1/ >1(%); ref=≤1) | 69(43.1)/91(56.9) | 23(52.3)/21(47.7) | 0.692(0.355-1.352) | 0.281 |  | |  |
| Intraop PRBC  (No/Yes(%);ref=No) | 78(48.8)/82(51.2) | 25(56.8)/19(43.2) | 0.723(0.369-1.416) | 0.344 |  | |  |
| Intraop FFP  ((No/Yes(%);ref=No) | 75(46.9)/85(53.1) | 20(45.5)/24(54.5) | 1.059(0.542-2.069) | 0.867 |  | |  |
| Operative time minutes  (≤360/ >360(%); ref=≤360) | 33(20.6)/127(79.4) | 6(13.6)/38(86.4) | 1.646(0.641-4.223) | 0.300 |  | |  |
| Time to surgery,days  (≤30/ >30(%); ref=≤30) | 49(30.6)/88(55.0) | 11(25.0)/29(65.9) | 1.468(0.675-3.193) | 0.333 |  | |  |
| LVI  (No/Yes(%);ref=No) | 119(74.4)/34(21.3) | 31(70.5)/11(25.0) | 1.242(0.566-2.727) | 0.589 |  | |  |
| MVI  (No/Yes(%);ref=No) | 102(63.7)/53(33.1) | 33(75.0)/9(20.5) | 1.012(0.984-1.041) | 0.393 |  | |  |
| R1 resection  ((R0/R2)/R1) (%); ref=R0/Rx) | 126(78.8)/32(20.0) | 36(81.8)/8(18.2) | 0.875(0.371-2.065) | 0.761 |  | |  |
| pT category  (T1-2/T3-4(%);ref=T1-T2) | 101(63.1)/57(35.6) | 32(72.7)/12(27.3) | 0.664(0.317-1.391) | 0.278 |  | |  |
| pN category  (N0/N1(%);ref=N0) | 81(50.6)/78(48.8) | 34(77.3)/10(22.7) | 0.305(0.141-0.660) | 0.003 | **0.307(0.123-0.767)** | | **0.012** |
| Tumor grading  ((G1/G2)/( G3/G4) (%);ref= G1/G2) | 104(65.0)/48(30.0) | 39(88.6)/5(11.4) | 0.278(0.103-0.749) | 0.011 | **0.248(0.076-0.808)** | | **0.021** |
| ICU time,days  (≤1/>1(%),ref=1） | 86(53.8)/74(46.3) | 26(59.1)/18(40.9) | 0.805(0.409-1.583) | 0.529 |  | |  |
| Hospitalization,days  (≤14/>14(%); ref=≤14) | 64(40.0)/96(60.0) | 20(45.5)/24(54.5) | 0.800(0.408-1.567) | 0.515 |  | |  |
| Postoperative complications Clavien-Dindo  ((0/I/II/IIIa)/(IIIa/IV/V) (%);ref= 0/I/II/IIIa) | 96(60.0)/64(40.0) | 24(54.5)/20(45.5) | 1.250(0.638-2.449) | 0.515 |  | |  |
| Liver failure  (No/Yes(%);ref=No) | 131(81.9)/29(18.1) | 36(81.8)/8(18.2) | 1.004(0.423-2.385) | 0.993 |  | |  |
| Bile leak  (No/Yes(%);ref=No) | 122(76.3)/38(23.8) | 33(75.0)/11(25.0) | 1.070(0.494-2.319) | 0.864 |  | |  |
| Hemorrhage  (No/Yes(%);ref=No) | 130(81.3)/30(18.8) | 35(79.5)/9(20.5) | 1.114(0.484-2.564) | 0.799 |  | |  |
| Infection Clavien-Dindo  ((0/I/II/IIIa)/(IIIa/IV/V) (%);ref=0/I/II/IIIa) | 123(76.9)/36(22.5) | 33(75.0)/11(25.0) | 1.139(0.524-2.477) | 0.743 |  | |  |
| Adjuvant therapy  (No/Yes(%);ref=No) | 110(68.8)/41(25.6) | 29(65.9)/13(29.5) | 0.971(0.826-1.141) | 0.719 |  | |  |
| **VSR** | **Low VSR(n=138)** | **High VSR(n=66)** | **OR (95% CI)** | **p-value** | **OR (95% CI)** | | **p-value** |
| Sex  (male/female(%); ref=male) | 75(54.3)/63(45.7) | 61(92.4)/5(7.6) | 0.098(0.037-0.258) | **＜0.001** | **0.075(0.027-0.206)** | | **＜0.001** |
| Age  (≤65/ >65 years(%); ref=≤65) | 66(47.8)/72(52.2) | 21(31.8)/45(68.2) | 1.964(1.061-3.638) | **0.032** | 2.439(1.213-4.904) | | **0.012** |
| ASA  ((I/II)/(III/IV) (%);ref= I/II) | 59(42.8)/79(57.2) | 18(27.3)/48(72.7) | 1.992(1.052-3.770) | **0.034** | 2.225(1.089-4.549) | | **0.028** |
| Cholangitis, (No/Yes(%);ref=No) | 93(67.4)/45(32.6) | 46(69.7)/20(30.3) | 0.899(0.477-1.694) | 0.741 |  | |  |
| PVE  (No/Yes(%);ref=No) | 84(60.9)/54(39.1) | 49(74.2)/17(25.8) | 0.543(0.282-1.033) | 0.063 |  | |  |
| Neoadjuvant therapy  ((No/Yes(%);ref=No) | 129(93.5)/9(6.5) | 65(98.5)/1(1.5) | 0.221(0.027-1.778) | 0.156 |  | |  |
| AST, U/L  (≤40/ >40(%); ref=≤40) | 59(42.8)/78(56.5) | 27(40.9)/36(54.5) | 1.009(0.552-1.843) | 0.978 |  | |  |
| ALT, U/L  (≤40/ >40(%); ref=≤40) | 5(3.6)/127(92.0) | 7(10.6)/58(87.9) | 0.981(0.494-1.951) | 0.957 |  | |  |
| AP,U/L  (≤100/ >100(%); ref=≤100) | 5(3.6)/127(92.0) | 7(10.6)/58(87.9) | 0.326(0.099-1.071) | 0.065 |  | |  |
| CRP,mg/l  (≤8.2/ >8.2(%); ref=≤8.2) | 55(39.9)/80(58.0) | 29(43.9)/37(56.1) | 0.795(0.433-1.461) | 0.460 |  | |  |
| GGT,U/L  (≤100/ >100(%); ref=≤100) | 9(6.5)/123(89.1) | 7(10.6)/58(87.9) | 0.606(0.215-1.708) | 0.344 |  | |  |
| Hemoglobin, g/L  (≤13/ >13(%); ref=≤13) | 97(70.3)/40(29.0) | 41(62.1)/25(37.9) | 1.479(0.796-2.746) | 0.215 |  | |  |
| INR  (≤1/>1(%); ref=≤1) | 55(39.9)/81(58.7) | 24(36.4)/42(63.6) | 1.188(0.647-2.181) | 0.578 |  | |  |
| Platelet count  (≤300/ >300(%); ref=≤300) | 73(52.9)/64(46.4) | 37(56.1)/29(43.9) | 0.894(0.495-1.614) | 0.710 |  | |  |
| Prothrombin time  (≤110/ >110(%); ref=≤110) | 113(81.9)/23(16.7) | 55(83.3)/11(16.7) | 0.983(0.447-2.160) | 0.965 |  | |  |
| Total bilirubin,mg/dl  (≤1/ >1(%); ref=≤1) | 64(46.4)/74(53.6) | 28(42.4)/38(57.6) | 1.174(0.649-2.121) | 0.596 |  | |  |
| Intraop PRBC  (No/Yes(%);ref=No) | 67(48.6)/71(51.4) | 36(54.5)/30(45.5) | 0.786(0.437-1.416) | 0.423 |  | |  |
| Intraop FFP  ((No/Yes(%);ref=No) | 58(42.0)/80(58.0) | 37(56.1)/29(43.9) | 0.568(0.314-1.027) | 0.061 |  | |  |
| Operative time minutes  (≤360/ >360(%); ref=≤360) | 29(21.0)/109(79.0) | 10(15.2)/56(84.8) | 1.490(0.678-3.275) | 0.321 |  | |  |
| Time to surgery,days  (≤30/ >30(%); ref=≤30) | 72(52.2)/51(37.0) | 30(45.5)/24(36.4) | 0.730(0.375-1.420) | 0.354 |  | |  |
| LVI  (No/Yes(%);ref=No) | 101(73.2)/30(21.7) | 49(74.2)/15(22.7) | 1.031(0.508-2.091) | 0.933 |  | |  |
| MVI  (No/Yes(%);ref=No) | 89(64.5)/45(32.6) | 46(89.7)/17(25.8) | 1.050(0.943-1.170) | 0.375 |  | |  |
| R1 resection  ((R0/R2)/R1) (%); ref=R0/Rx) | 108(78.3)/29(21.0) | 54(81.8)/11(16.7) | 0.759(0.352-1.634) | 0.480 |  | |  |
| pT category  (T1-2/T3-4(%);ref=T1-T2) | 91(65.9)/46(33.3) | 42(63.6)/23(34.8) | 1.083(0.583-2.014) | 0.800 |  | |  |
| pN category  (N0/N1(%);ref=N0) | 78(56.5)/60(43.5) | 37(56.1)/28(42.4) | 0.984(0.542-1.784) | 0.957 |  | |  |
| Tumor grading  ((G1/G2)/( G3/G4) (%);ref= G1/G2) | 91(65.9)/40(29.0) | 52(78.8)/13(19.7) | 0.569(0.279-1.160) | 0.121 |  | |  |
| ICU time,days  (≤1/>1(%),ref=1） | 76(55.1)/62(44.9) | 36(54.5)/30(45.5) | 1.022(0.567-1.841) | 0.944 |  | |  |
| Hospitalization,days  (≤14/>14(%); ref=≤14) | 54(39.1)/84(60.9) | 30(45.5)/36(54.5) | 0.771(0.426-1.396) | 0.391 |  | |  |
| Postoperative complications Clavien-Dindo  ((0/I/II/IIIa)/(IIIa/IV/V) (%);ref= 0/I/II/IIIa) | 79(57.2)/59(42.8) | 41(62.1)/25(37.9) | 0.816(0.448-1.489) | 0.508 |  | |  |
| Liver failure  (No/Yes(%);ref=No) | 112(81.2)/26(18.8) | 55(83.3)/11(16.7) | 0.862(0.397-1.871) | 0.706 |  | |  |
| Bile leak  (No/Yes(%);ref=No) | 107(77.5)/31(22.5) | 48(72.7)/18(27.3) | 1.294(0.660-2.538) | 0.453 |  | |  |
| Hemorrhage  (No/Yes(%);ref=No) | 105(76.1)/33(23.9) | 60(90.9)/6(9.1) | 0.318(0.126-0.803) | **0.015** | 0.274(0.101-0.742) | | **0.011** |
| Infection Clavien-Dindo  ((0/I/II/IIIa)/(IIIa/IV/V) (%);ref=0/I/II/IIIa) | 101(73.2)/37(26.8) | 55(83.3)/10(15.2) | 0.496(0.229-1.074) | 0.075 |  | |  |
| Adjuvant therapy  (No/Yes(%);ref=No) | 98(71.0)/35(25.4) | 41(62.1)/19(28.8) | 0.971(0.857-1.101) | 0.649 |  | |  |

Multiple variables were associated with body composition. Variables displaying a p value < 0.05 in the univariate analysis were transferred into a multivariable logistic regression model.

Abbreviations: ALT, alanine aminotransferase; AP, Alkaline phosphatase; ASA, American Society of Anesthesiologists; AST, aspartate aminotransferase; BMI, body mass index; CRP, C-reactive protein; FFP, Fresh frozen plasma; GGT, gamma-glutamyl transferase; OR, Odds ratio; ICU, intensive care unit; INR, international normalized ratio; LVI, lymph vascular invasion; MVI, microvascular invasion; PRBC, Packed Red Blood Cells; PVE, portal vein embolization; **VSR**, visceral to subcutaneious adipose tissue ratio.
